# Loss-of-function variants in *ODAD1* disrupt ODA docking and induce actin cytoskeletal remodeling in primary ciliary dyskinesia

**DOI:** 10.1038/s41421-026-00875-8

**Published:** 2026-03-31

**Authors:** Chunxiao Huo, Ting Luo, Shuo Yang, Yuan Jiang, Mengzhe Guo, Feng Yang, Zhangqi Xu, Junhua Xia, Lei Wu, Weize Xu, Miao Gui, Tianhua Zhou, Shanshan Xie

**Affiliations:** 1 Children’s Hospital, Zhejiang University School of Medicine, National Clinical Research Center for Children and Adolescents’ Health and Diseases, Hangzhou, Zhejiang, China; 2 Binjiang Institute of Zhejiang University, Hangzhou, Zhejiang, China; 3 Department of Cell Biology, Zhejiang University School of Medicine, Hangzhou, Zhejiang, China; 4 Department of Obstetrics and Gynecology, Sir Run Run Shaw Hospital and Liangzhu Laboratory, Zhejiang University School of Medicine, Hangzhou, Zhejiang, China; 5 Department of Pulmonology, Children’s Hospital, Zhejiang University School of Medicine, National Clinical Research Center for Children and Adolescents’ Health and Diseases, Hangzhou, Zhejiang, China; 6 Jiangsu Key Laboratory of New Drug Research and Clinical Pharmacy, Xuzhou Medical University, Xuzhou, Jiangsu, China; 7 Department of Endoscopy Center, Children’s Hospital, Zhejiang University School of Medicine, National Clinical Research Center for Children and Adolescents’ Health and Diseases, Hangzhou, Zhejiang, China; 8 Zhejiang Key Laboratory of Neonatal Diseases, Hangzhou, Zhejiang, China

**Keywords:** Mechanisms of disease, Organelles

## Abstract

Primary ciliary dyskinesia (PCD) is a genetically heterogeneous disorder characterized by defective mucociliary clearance due to impaired motile cilia function. *Outer dynein arm docking complex subunit 1* (*ODAD1*) encodes a structural component of the outer dynein arm docking complex (ODA-DC), which is crucial for effective ciliary beating. However, the full spectrum of *ODAD1*-associated pathogenesis remains incompletely defined. Here, we identified one novel homozygous loss-of-function *ODAD1* variant, c.705_706insGCAG, and a recurrent homozygous splicing variant, c.-41-2A > C, in seven unrelated patients. These patients exhibited hallmark PCD symptoms with reduced nasal nitric oxide levels and situs inversus. Patient-derived nasal epithelial cells and air‒liquid interface (ALI) cultures exhibited a markedly reduced ciliary beating frequency and severe motility defects. Transmission electron microscopy and cryo-electron microscopy showed a complete loss of outer dynein arms and docking complexes, with variant-specific ultrastructural abnormalities. Unexpectedly, *ODAD1* deficiency also led to reduced multiciliated cells (MCCs) abundance, misoriented basal bodies, and impaired multiciliogenesis. Proteomic profiling and immunostaining revealed prominent actin cytoskeletal remodeling, including aberrant F-actin bundling throughout the epithelial layers. Pharmacological inhibition of actin polymerization using cytochalasin B partially rescued the abundance of MCCs and multiciliogenesis, indicating that actin dysregulation is a modifiable consequence of *ODAD1* loss. Finally, lentiviral re-expression of wild-type *ODAD1* in patient-derived organoids restored ODA assembly and rescued coordinated ciliary beating, confirming the pathogenicity of the identified variants. Our data reveal a dual role for ODAD1 in regulating both axonemal structure and epithelial cytoskeletal integrity and identify actin dysregulation as a previously unrecognized, targetable pathological mechanism of *ODAD1*-associated PCD.

## Introduction

Motile cilia on the epithelial surfaces of the respiratory tract are essential for airway clearance through their coordinated beating, which propels mucus and entrapped particles out of the lung^1^. This function is disrupted in patients with primary ciliary dyskinesia (PCD), a rare, genetically diverse disorder characterized by defective motile cilia that leads to chronic respiratory infections, rhinosinusitis, bronchiectasis, and variably situs abnormalities^2^. The estimated global prevalence of PCD is at least 1 in 7554 individuals^3^. PCD is predominantly inherited in an autosomal recessive manner, with over 50 pathogenic genes, many of which encode components of the cilia axoneme or its regulatory machinery^3^.

Among the key structural modules of motile cilia, the outer dynein arms (ODAs) are responsible for generating the mechanical force required for ciliary beating. These ODAs are anchored to the axoneme — a conserved microtubule-based core composed of nine doublet microtubules (DMTs) arranged in a circular pattern — through the outer dynein arm-docking complex (ODA-DC). ODA-DC comprises several essential proteins, including ODAD1 (also known as CCDC114), ARMC4 (ODAD2), CCDC151 (ODAD3), TTC25 (ODAD4), and Calaxin (CLXN), and facilitates the stable attachment of ODAs to DMTs^4^. ODAD1, a coiled-coil domain-containing protein, plays a pivotal role in maintaining the integrity of this complex and securing ODAs to the axoneme^5–7^. Disruption of ODA-DC components, as observed in PCD patients carrying variants in *ODAD1* or related genes, leads to the loss or mislocalization of ODAs and impaired ciliary motility^5–12^. In addition to ODAs, other axonemal substructures, including inner dynein arms (IDAs), radial spokes (RSs), nexin–dynein regulatory complexes (N-DRCs), and microtubule inner proteins (MIPs), work in concert to regulate ciliary movement^13^. While the structural role of ODAD1 in dynein arm docking is well characterized, its broader effects on axonemal integrity and cellular function remain largely unexplored.

While *ODAD1* variants are an established cause of PCD^5,11^, the mutational spectrum remains incompletely defined, particularly in non-European populations. In this study, we identified a novel homozygous *ODAD1* variant (c.705_706insGCAG) and a recurrent variant (c.-41-2A > C) in seven Han Chinese families with PCD. Using patient-derived nasal epithelial cells and air–liquid interface (ALI) cultures, we show that these variants abolish ODA assembly, resulting in defective ciliary motility. Unexpectedly, *ODAD1* deficiency also reduced the number of multiciliated cells (MCCs) and impaired multiciliogenesis, which we traced to dysregulated actin cytoskeletal remodeling. Importantly, pharmacological inhibition of actin polymerization partially restored the abundance of MCCs and promoted ciliogenesis. Together, our findings both expand the spectrum of *ODAD1* variants in patients with PCD and reveal a previously unrecognized link between *ODAD1* dysfunction and actin regulation, highlighting actin remodeling as a potential therapeutic target.

## Results

### Identification of novel and recurrent *ODAD1* variants associated with PCD

**Fig. 1:**
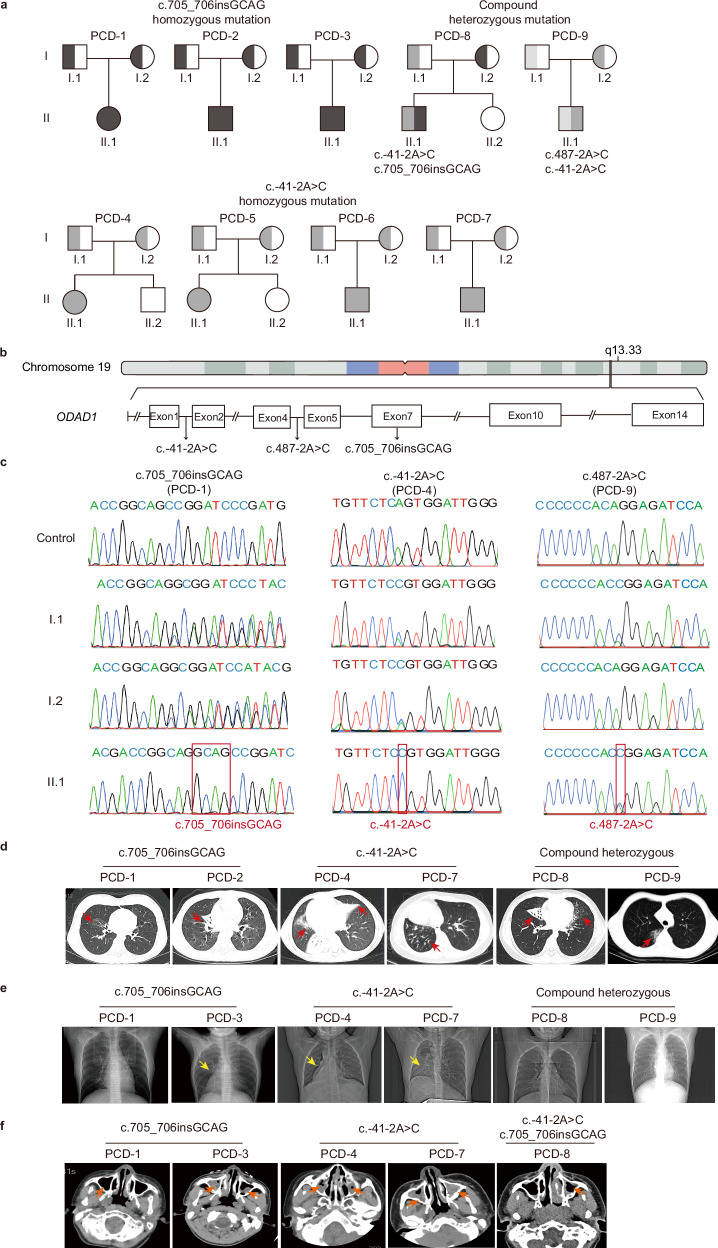
Identification of pathogenic *ODAD1* variants in patients with PCD. **a** Pedigrees of nine unrelated Chinese families with *ODAD1* variants. PCD-1 to PCD-3 harbor the homozygous variant c.705_706insGCAG. PCD-4 to PCD-7 harbor the homozygous variant c.-41-2A > C. Family PCD-8 carried compound heterozygous variants (c.-41-2A > C and c.705_706insGCAG), whereas family PCD-9 carried compound heterozygous variants (c.487-2 A > C and c.-41-2A > C). **b** Schematic overview showing the location of *ODAD1* (Gene ID: 93233) on chromosome 19q13.33. **c** Sanger sequencing chromatograms for families PCD-1 (left panel), PCD-4 (middle panel) and PCD-9 (right panel). **d**–**f** Representative high-resolution computed tomography (HRCT) scans of the chest and nasal sinuses of patients with *ODAD1*-variant PCD, showing bronchiectasis, mucus plugging, situs inversus and nasosinusitis. Red arrows indicate pulmonary consolidation (**d**), yellow arrows indicate situs inversus (**e**), and orange arrows indicate nasosinusitis (**f**).

A genetic analysis identified four distinct pathogenic *ODAD1* variants (reference transcript: NM_144577.4) in nine individuals with PCD (Fig. 1a). Three patients from unrelated families carried a homozygous frameshift variant, c.705_706insGCAG, which represents a novel variant not previously reported in population databases. Four patients were homozygous for the splice-site variant c.-41-2A > C (equivalent to c.71-2 A > C on NM_001364171.2; GRCh38:chr19:g.48318814 A > C), which has been previously associated with PCD in ClinVar (VCV003383099.1) but is reported here with detailed phenotypic characterization for the first time. Two additional patients harbored compound heterozygous variants: one with c.-41-2A > C and c.705_706insGCAG, and the other with c.487-2 A > C and c.-41-2A > C. An analysis of the pedigree confirmed autosomal recessive inheritance, with both parents in each family being heterozygous carriers (Fig. 1b, c). Notably, the recurrence of the identified *ODAD1* variants (c.705_706insGCAG and c.-41-2A > C) in our patient cohort may be indicative of a potential founder effect rather than reflecting the general population frequency.

Clinically, all nine patients presented typical PCD features, such as chronic cough, rhinosinusitis, recurrent respiratory infections, and bronchiectasis (Table 1 and Fig. 1d–f), along with low nasal nitric oxide levels (8.4–31.0 ppb), which were significantly lower than those in healthy Chinese individuals aged 6–18 years (454.5 ± 176.2 ppb)^14^. Patients homozygous for the c.705_706insGCAG variant experienced neonatal respiratory distress and developed recurrent infections complicated by bronchiectasis, atelectasis, and otitis media. Similarly, those homozygous for the c.-41-2A > C variant also experienced recurrent infections, with three individuals developing bronchiectasis. Patients with compound heterozygous variants likewise exhibited chronic respiratory infections and progression to bronchiectasis, with an absence of otitis media. Situs inversus was observed in several patients harboring *ODAD1* variants, consistent with the randomization of left–right body asymmetry (Fig. 1e and Table 1). Notably, subtle extra-respiratory findings, including nutcracker syndrome (PCD-1 and PCD-2) and ventriculomegaly (PCD-3), were observed only in patients with the c.705_706insGCAG variant, whereas no such abnormalities were documented in individuals carrying the c.-41-2A > C variant.

**Table 1:** Clinical manifestations of PCD patients with *ODAD1* variants.

Characteristic	PCD-1	PCD-2	PCD-3	PCD-4	PCD-5	PCD-6	PCD-7	PCD-8	PCD-9
Onset age (y)	0	5	0	0	NA	0	0	0	0
Diagnosis age (y)	13	10	8	6	NA	0	22	12	10
Age (y)	13	14	13	6	28	1	22	13	11
Sex	Female	Male	Male	Female	Female	Male	Male	Male	Male
Neonatal respiratory distress	Yes	No	Yes	Yes	NA	Yes	Yes	Yes	Yes
Recurrent respiratory infections	Yes	Yes	Yes	Yes	Yes	Yes	Yes	Yes	Yes
Bronchiectasis	Yes	Yes	Yes	Yes	Yes	No	Yes	Yes	Yes
Atelectasis	Yes	Yes	Yes	Yes	No	Yes	Yes	Yes	Yes
Sinusitis	Yes	Yes	Yes	Yes	Yes	Yes	Yes	Yes	Yes
Otitis media	Yes	Yes	Yes	Yes	No	Yes	Yes	No	No
Situs inversus	No	No	Yes	Yes	Yes	Yes	Yes	No	No
Cardiac abnormalities	No	Yes	No	Yes	No	No	No	Yes	No
Ventricular abnormalities	No	No	Yes	No	No	No	No	No	No
Nutcracker syndrome	Yes	Yes	No	No	No	No	No	No	No
FeNO_50_ (ppb)	3.1	–	4.5	–	–	–	7.6	3.6	7
FnNO_10_ (ppb)	23.2	–	8.4	–	–	–	19.6	12.6	31

Data are presented as n or median (IQR). FeNO_50_: fractional exhaled nitric oxide at 50 mL/s; FnNO_10_: fractional nasal nitric oxide at 10 mL/s.

Reverse transcription polymerase chain reaction (RT‒PCR) indicated that both the *ODAD1* homozygous variants c.705_706insGCAG and c.-41-2A > C resulted in decreased *ODAD1* expression levels in patients’ nasal epithelial cells (Supplementary Fig. S1a–c). Sanger sequencing revealed that the c.705_706insGCAG variant introduced a tetranucleotide insertion (GCAG) between nucleotides 705 and 706 (Supplementary Fig. S1d), whereas the c.-41-2A > C variant caused aberrant splicing, leading to the deletion of the first 19 nucleotides of exon 2 (Supplementary Fig. S1e). As the deletion occurs upstream of the start codon, it may interfere with translation initiation.

We modeled the amino acid sequence of the protein encoded by the c.705_706insGCAG variant transcript to examine the effects of the variant on the protein product. The resulting protein retained 247 amino acids, including the two conserved coiled-coil domains, but was truncated compared with the full-length wild-type protein (670 amino acids) (Supplementary Fig. S1f, g). Flag-tagged wild-type and variant constructs were expressed in hRPE-1 cells to experimentally assess their expression, and truncated proteins (~30–35 kDa) were produced from the variant (Supplementary Fig. S1h). Immunostaining of patient-derived nasal epithelial cells showed a loss of the endogenous ODAD1 protein (Supplementary Fig. S1i), confirming the loss-of-function consequences of these variants. The faint, diffuse cytoplasmic signal observed in cells from some patients is consistent with non-specific background staining and/or the presence of unstable truncated protein products that are incapable of functional ciliary incorporation. Taken together, the near-complete absence of the mature *ODAD1* transcript and protein in c.-41-2A > C homozygous samples strongly supports the classification of this splice-site mutation as a loss-of-function *ODAD1* allele.

### *ODAD1* variants disrupt ciliary motility in PCD patients

**Fig. 2:**
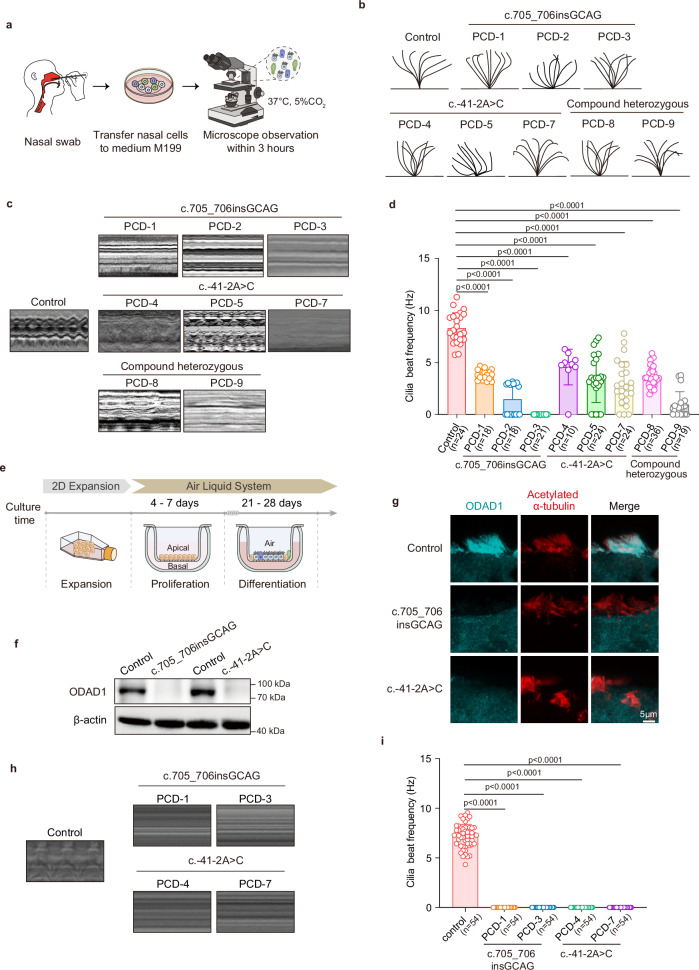
Analysis of ciliary motility in patients harboring *ODAD1* variants. **a** Schematic workflow of the HSVM analysis, including nasal swab collection, medium transfer, and imaging. **b** Ciliary beat pattern (CBP) of nasal epithelial cells from a healthy control and patients harboring *ODAD1* variants. **c** Kymography analysis of ciliary beating in samples from healthy controls and patients carrying the variants. **d** Quantification of the CBF of nasal epithelial cells from a healthy control and patients carrying *ODAD1* variants. Each data point represents the CBF measured from one ciliary bundle. The data are derived from two independent samples per individual. The total number (*n*) of bundles analyzed for each group is indicated on the graph. **e** Schematic diagram of ALI cultures of human nasal epithelial cells. **f** Western blot analysis of th levels of the ODAD1 protein in ALI cultures from a healthy control and patients carrying *ODAD1* variants. **g** Immunofluorescence staining for acetylated α-tubulin (red) and ODAD1 (cyan) in ALI cultures from healthy controls and patients carrying *ODAD1* variants. Scale bar, 5 μm. **h** Kymography analysis of ciliary beating in ALI cultures from healthy controls and patients carrying *ODAD1* variants. **i** Quantification of CBF in ALI cultures from a healthy control and patients carrying *ODAD1* variants. Each data point represents the CBF measured from one ciliary bundle. The data are derived from three independent samples per individual. The total number (*n*) of bundles analyzed for each group is indicated on the graph. **f**–**i** ALI cultures were analyzed on Day 24 of differentiation. **d**, **i** *P* values were determined using one-way ANOVA with Tukey’s multiple comparison test and are indicated directly in the figures. Data are presented as means ± SEM.

Given the essential role of ODAD1 in ciliary motility, we performed high-speed video microscopy (HSVM) on freshly isolated nasal epithelial cells from affected individuals (Fig. 2a and Supplementary Videos S1–S9). In contrast to the coordinated, rhythmic beating observed in healthy controls, motile cilia from patients carrying the *ODAD1* variants displayed irregular, uncoordinated movement (Fig. 2b). A kymography analysis confirmed the loss of the typical sine-wave pattern, indicating severe mechanical dysfunction (Fig. 2c). The quantitative analysis revealed a marked reduction in the ciliary beat frequency (CBF) (Fig. 2d), which was consistent with impaired mucociliary clearance and chronic respiratory symptoms.

We established air‒liquid interface (ALI) cultures from patient-derived nasal epithelial cells to further assess the functional impact of these variants. These cultures recapitulated the architecture and function of the airway epithelium^15–17^ (Fig. 2e). Western blot and immunostaining analyses showed the complete absence of the full-length ODAD1 protein in cultures from patients homozygous for the c.705_706insGCAG or c.-41-2A > C variant, with a corresponding loss of its localization to motile cilia (Fig. 2f, g). Consistent with the observations in primary cells, the HSVM of variant ALI cultures showed severely compromised ciliary motility, with disorganized, low-amplitude beating (Fig. 2h and Supplementary Videos S10–S14). A quantitative analysis of the CBF confirmed significantly reduced ciliary activity in the ALI cultures carrying variants compared with wild-type controls (Fig. 2i), reinforcing the critical role of ODAD1 in maintaining effective ciliary motion. We observed that the ciliary beat pattern differed between freshly isolated nasal brushings and ALI cultures. Nasal brushings contain a heterogeneous cell mixture and mucus, where partially differentiated or mechanically stimulated cilia can show limited residual movement. In contrast, ALI cultures generate a uniform, fully differentiated epithelium that unmasks the intrinsic immotility caused by *ODAD1* dysfunction.

### Structural defects are present in *ODAD1*-variant cilia from ALI cultures

**Fig. 3:**
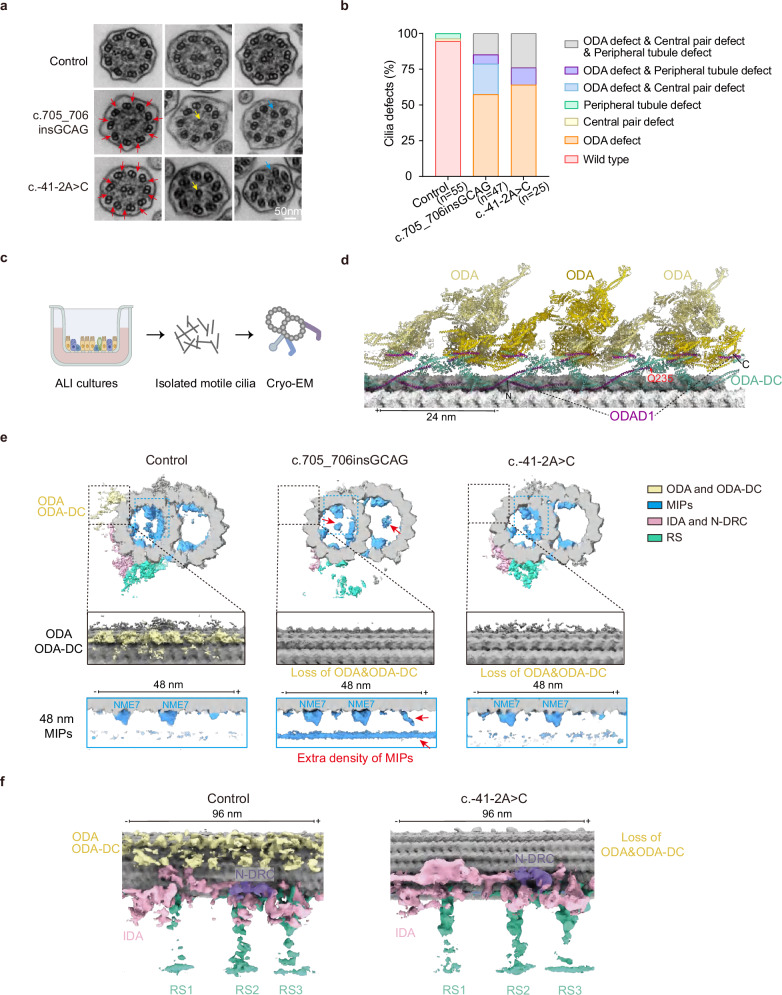
Structural analysis of ciliary defects in *ODAD1*-variant ALI cultures. **a**, **b** TEM of cilia from control and *ODAD1*-variant ALI cultures on Day 24 of ALI differentiation. **a** Representative ciliary cross-sections. **b** Categorization of ultrastructural defects from multiple cross-sections. Quantification was based on an analysis of 55 cross-sections from 3 control individuals (1 culture each), 47 cross-sections from 2 patients homozygous for c.705_706insGCAG (PCD-1: 22 cross-sections; PCD-3: 25 cross-sections; 1 culture each), and 25 cross-sections from 2 patients homozygous for c.-41-2A > C (PCD-4: 11 cross-sections; PCD-7: 14 cross-sections; 1 culture each). Red arrows indicate ODA defects, yellow arrows indicate central pair defects, and blue arrows represent misplaced DMTs. **c** Cryo-EM workflow of the axoneme analysis. **d** Atomic model of the human respiratory axoneme showing the structure of ODAD1 (PDB: 8J07). The Q235 insertion site of c.705_706insGCAG in the patient is indicated by the red arrow. **e** Cryo-EM density maps of 48-nm DMTs from control and *ODAD1*-variant cultures. Axonemal complexes are indicated with different colors. Insets of longitudinal views showing the loss of ODA and ODA-DC in both variants. The MIP organization in cultures expressing the c.-41-2A > C variant resembles that in the control cultures, whereas the MIP in cultures expressing the c.705_706insGCAG variant contains extra densities. Red arrows indicate abnormal densities in the cultures expressing the c.705_706insGCAG variant, including an extra density in the center of the A-tubule, extra density in the center of the B-tubule, and disrupted density near the NME7 landmark. **f** Cryo-EM density maps of 96-nm DMTs from the control cultures and cultures expressing the c.-41-2A > C variant show the loss of ODA and ODA-DC and the preservation of IDA, RS, and N-DRC. All cryo-EM analyses were performed on axonemes isolated from ALI cultures on Day 24 of differentiation.

We investigated whether *ODAD1* variants (c.705_706insGCAG and c.-41-2A > C) lead to ultrastructural abnormalities by performing transmission electron microscopy (TEM) on motile cilia derived from ALI cultures on Day 24 of differentiation. The TEM analysis revealed classic PCD-associated defects, including the complete loss of ODAs, central pair (CP) abnormalities, and peripheral tubule (PT) defects (Fig. 3a, b). Immunofluorescence staining for the CP marker SPEF2 further showed a marked reduction in SPEF2-positive ciliary tufts in *ODAD1*-deficient ALI cultures (Supplementary Fig. S3a, b), confirming the presence of a previously unreported CP defect.

We isolated motile cilia from ALI cultures of a healthy control and two patients homozygous for the *ODAD1* variants (PCD-1: c.705_706insGCAG and PCD-4: c.-41-2A > C) to further assess these structural defects at near-atomic resolution. Following detergent treatment to remove the ciliary membrane, axonemes were analyzed using cryo-electron microscopy (cryo-EM) (Fig. 3c, d). The initial 3D refinement of 8-nm particles yielded clear density maps of DMTs from both control and variant cilia (Supplementary Fig. S2a, b). Three-dimensional classification was performed to generate DMT particles with 48 nm and 96 nm periodicities and resolve the MIPs and axonemal complexes (Supplementary Fig. S2c–e and Table S1). Both variants exhibited a complete loss of the ODA and ODA-DC, suggesting that ODAD1 is essential for ODA docking (Fig. 3e). MIPs, IDA, RS and N-DRC in the c.-41-2A > C-variant cilia retained structural integrity compared with those in the controls (Fig. 3e, f). Notably, in the c.705_706insGCAG-variant cilia, the 48-nm density map generated using the NME7 globular domain as a classification marker revealed an extra density within both A-tubules and B-tubules as well as a disrupted density near the NME7 landmark, implying imperfect MIP assembly. Furthermore, while axonemal complexes were detected, 3D classification failed to reconstruct a 96-nm density map, indicating the potential mislocalization of IDA, RS, and N-DRC in the c.705_706insGCAG-variant cilia (Fig. 3e).

Collectively, our TEM and cryo-EM analyses demonstrate that distinct *ODAD1* variants lead to the complete loss of the ODA and ODA-DC, as well as varying degrees of CP loss, mislocalized DMTs, and impaired docking of MIPs and axonemal complexes, highlighting the critical role of ODAD1 in maintaining the ciliary architecture and motility. Intriguingly, structural analyses showed that distinct *ODAD1* variants exert different effects on the axonemal architecture.

### *ODAD1* variants reduce the abundance of MCCs and disrupt their apical organization

**Fig. 4:**
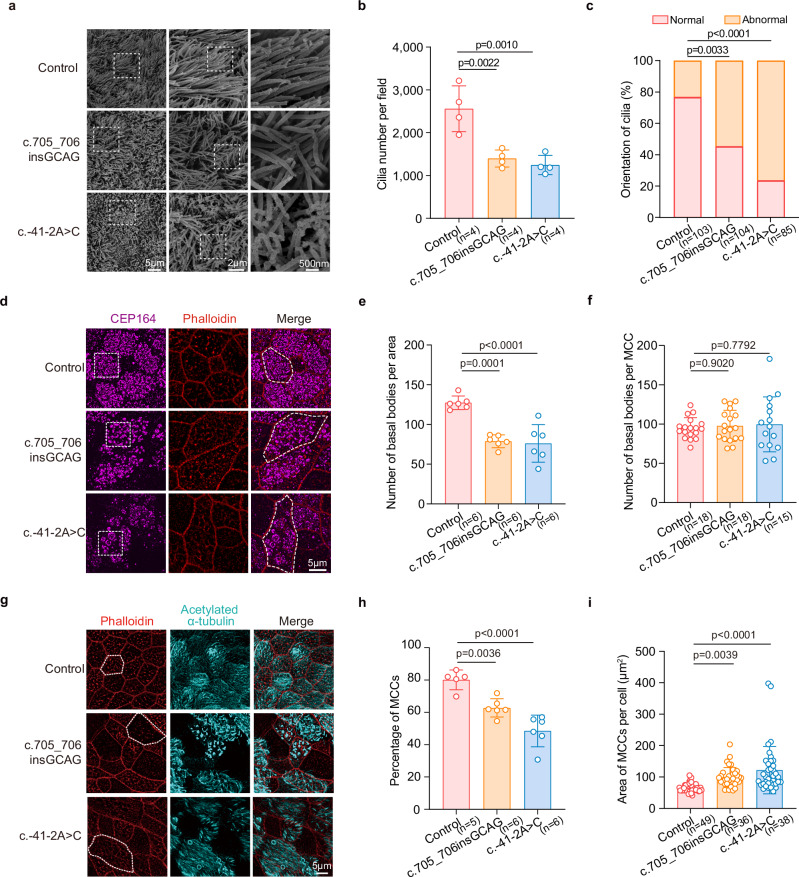
MCC-related phenotypic analysis in *ODAD1*-variant ALI cultures. **a**–**c** SEM images of cilia in ALI cultures. **a** Representative images of the ciliary morphology. **b** Cilia number per field. Each data point represents the cilium count from one SEM field of view (3,000× magnification). The data were obtained from 2 control individuals (2 fields per individual; total *n* = 4 fields), 2 patients homozygous for c.705_706insGCAG (PCD-1: 2 fields; PCD-3: 2 fields; total *n* = 4 fields), and 2 patients homozygous for c.-41-2A > C (PCD-4: 2 fields; PCD-7: 2 fields; total *n* = 4 fields). **c** Quantitative analysis of the ciliary orientation. The percentage of fields with normal and abnormal ciliary orientations is shown. Quantification was based on the analysis of 103 fields from 2 control individuals (41 and 62 fields), 104 fields from 2 patients carrying the c.705_706insGCAG variant (PCD-1: 54 fields; PCD-3: 50 fields), and 85 fields from 2 patients carrying the c.-41-2A > C (PCD-4: 44 fields; PCD-7: 41 fields). **d**–**f** Immunofluorescence staining for CEP164 (magenta) and phalloidin (red) in ALI cultures. **d** Representative images. **e** Number of basal bodies per area. Each data point represents the basal body count within a standardized area from one randomly acquired confocal field of view. The data were obtained from 2 control individuals (3 fields per individual; total *n* = 6), 2 patients homozygous for c.705_706insGCAG (PCD-1: 3 fields; PCD-3: 3 fields; total *n* = 6), and 2 patients homozygous for c.-41-2A > C (PCD-4: 3 fields; PCD-7: 3 fields; total *n* = 6). **f** Number of basal bodies per MCC. Each data point represents the basal body count within an individual MCC. The data were obtained from 2 control individuals (9 MCCs per individual; total *n* = 18), 2 patients homozygous for c.705_706insGCAG (PCD-1: 9 MCCs; PCD-3: 9 MCCs; total *n* = 18), and 2 patients homozygous for c.-41-2A > C (PCD-4: 7 MCCs; PCD-7: 8 MCCs; total *n* = 15). **g**–**i** Immunofluorescence staining for acetylated α-tubulin (cyan) and phalloidin (red) in ALI cultures. **g** Representative images. **h** Percentage of MCCs. Each data point represents the percentage of MCCs within one randomly acquired confocal image. The data were obtained from 2 control individuals (2 and 3 images per individual; total *n* = 5), 2 patients homozygous for c.705_706i*n*sGCAG (PCD-1: 3 images; PCD-3: 3 images; total *n* = 6), and 2 patients homozygous for c.-41-2A > C (PCD-4: 3 images; PCD-7: 3 images; total *n* = 6). **i** Area of MCCs per cell. Each data point represe*n*ts the apical surface area of an individual MCC. The data were obtained from 2 control individuals (24 and 25 MCCs per individual; total *n* = 49), 2 patients homozygous for c.705_706insGCAG (PCD-1: 18 MCCs; PCD-3: 18 MCCs; total *n* = 36), and 2 patients homozygous for c.-41-2A > C (PCD-4: 19 MCCs; PCD-7: 19 MCCs; total *n* = 38). All experiments were performed on Day 24 of ALI differentiation, with the data collected from two independent culture batches. *P* values were determined using one-way ANOVA with Tukey’s multiple comparison test and are indicated directly in the figures. Data are presented as means ± SEM.

Scanning electron microscopy (SEM) of *ODAD1*-variant ALI cultures at differentiation Day 24 revealed a pronounced reduction in the ciliary density, accompanied by misoriented cilia, compared with those of the controls (Fig. 4a–c). We assessed the distribution and density of basal bodies, which are critical for proper cilia formation and alignment^18^, to explore the structural basis of these defects. Structured illumination microscopy (SIM) revealed a significant reduction in the basal body density per unit area in *ODAD1*-variant cultures (Fig. 4d, e), along with an irregular apical distribution lacking the uniform spacing and orientation observed in the healthy epithelium.

We quantified the number of basal bodies per MCC to determine whether the observed decrease in basal body density was due to impaired basal body biogenesis or a reduction in MCCs abundance. This number was comparable between control and *ODAD1*-variant cultures (Fig. 4f), indicating that basal body generation within individual MCCs was unaffected. Instead, the overall reduction in the basal body density resulted from a decreased proportion of MCCs in the *ODAD1*-variant epithelium, as confirmed by immunofluorescence staining and confocal imaging (Fig. 4g, h). In addition, individual MCCs displayed a significantly enlarged apical surface area (Fig. 4i), suggesting altered epithelial packing and polarity rather than delayed basal body amplification.

We extended the ALI cultures to Day 36 to exclude the possibility that the observed defects were due to delayed differentiation*. ODAD1*-variant ALI cultures continued to display reduced MCCs numbers (Supplementary Fig. S4a, b), enlarged MCCs apical surface areas (Supplementary Fig. S4a, c), and abnormal basal body distributions (Supplementary Fig. S4d–f), consistent with the persistent nature of these phenotypes.

Together, these results indicate that *ODAD1* variants compromise the epithelial architecture primarily by reducing MCCs abundance and disrupting their apical organization, whereas basal body biogenesis per MCC remains intact.

### *ODAD1* variants result in actin cytoskeletal remodeling in MCCs

**Fig. 5:**
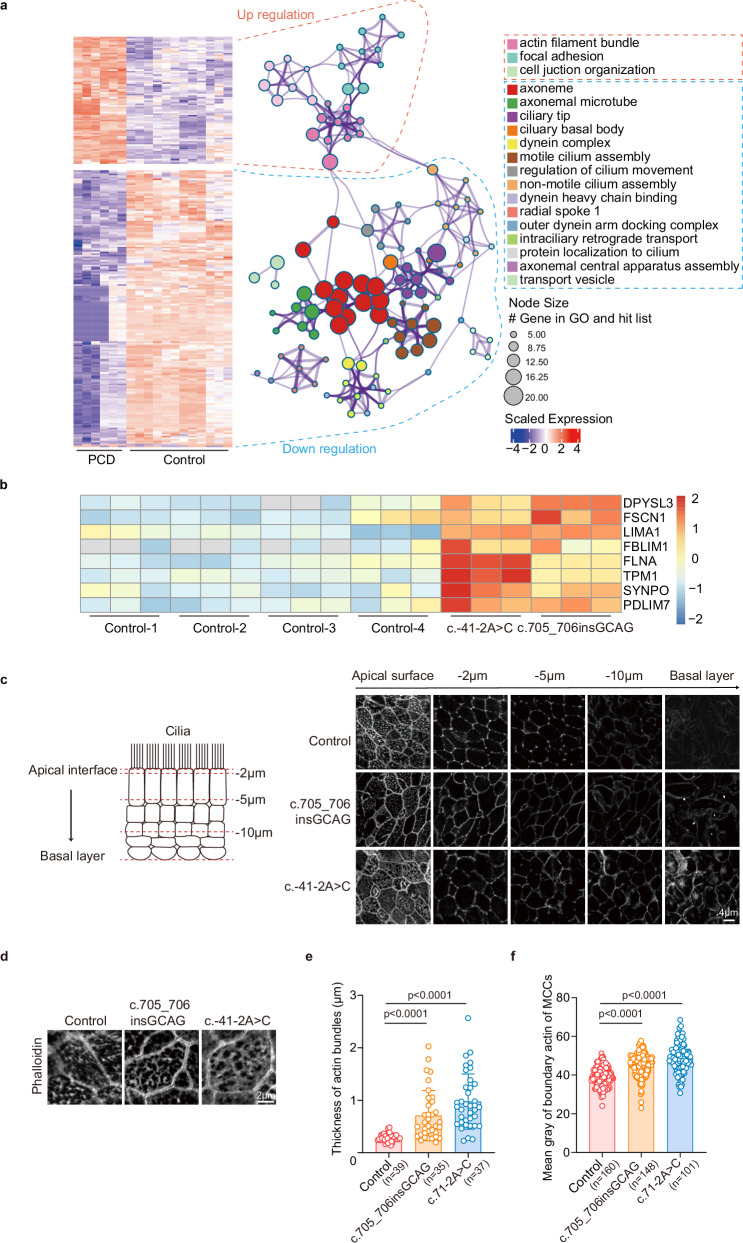
*ODAD1* variants lead to actin cytoskeletal dysregulation. **a** GO enrichment analysis of differentially expressed proteins in *ODAD1*-variant ALI cultures compared with controls on Day 24 of ALI differentiation. **b** Heatmap showing the expression of actin-associated proteins in ALI cultures. **c** Representative confocal z-projections (side views) of ALI cultures stained for F-actin, showing the distribution from the apical interface to the basal layer in control and *ODAD1*-variant ALI cultures. **d**–**f** Immunofluorescence staining for F-actin in MCCs in control and *ODAD1*-variant ALI cultures. High-sensitivity structured illumination microscopy (HIS-SIM) images of the apical surface of MCCs, showing F-actin staining in control and *ODAD1*-variant cultures (**d**). Quantification of the actin bundle thickness. Scatter plot showing the full width at half maximum (FWHM) of individual actin bundles measured from HIS-SIM images. Each point represents a single-bundle measurement. The data were derived from three independent experiments. The total number of bundles measured (*n*) for each genotype is indicated on the *x* axis (**e**). Quantification of the boundary actin intensity (gray value) in MCCs. Each point represents the averaged boundary actin intensity from a single MCC. The total number of MCCs measured (*n*) for each genotype is indicated on the *x* axis (**f**). All the data were pooled from three independent biological replicates. **e**, **f** *P* values were determined using one-way ANOVA with Tukey’s multiple comparison test and are indicated directly in the figures. Data are presented as means ± SEM.

We investigated the molecular mechanisms underlying MCC defects in *ODAD1*-variant cultures by performing a quantitative proteomic analysis on ALI cultures derived from patients carrying the homozygous c.705_706insGCAG or c.-41-2A > C variants. The differential expression analysis ( | log_2_(fold change)|> 0.5; adj. *P* < 0.05) identified 80 consistently upregulated proteins and 168 downregulated proteins across both *ODAD1*-variant cultures (Supplementary Fig. S5a and Tables S2–S3). The Gene Ontology (GO) enrichment analysis revealed that downregulated proteins were significantly associated with ciliary components and functions, including the axoneme, basal body, motile cilium assembly and regulation of ciliary movement (Supplementary Fig. S5b), whereas upregulated proteins were enriched for actin cytoskeleton-related processes, such as filament bundle assembly, and actin organization and dynamics (Fig. 5a–c and Supplementary Tables S4 and S5).

Prompted by these findings, we examined the actin architecture using F-actin staining. The *ODAD1*-variant cultures exhibited marked cytoskeletal remodeling across all the epithelial layers (Fig. 5c). At the apical surface, compared with the MCCs in the control cultures, the MCCs in the *ODAD1*-variant cultures displayed significantly thicker and denser F-actin bundles. In the middle layer, the actin filaments of the *ODAD1*-variant cultures were hypercompacted along cell borders; furthermore, in the basal layer, the normal filamentous actin network was replaced by patchy, punctate aggregates. A quantitative analysis of the images confirmed the increased actin bundle thickness and elevated F-actin intensity at cell junctions in *ODAD1*-variant MCCs (Fig. 5d–f), indicating aberrant actin bundling and polymerization.

We generated CRISPR/Cas9-mediated *ODAD1* knockout ALI cultures from healthy donor airway epithelial cells to exclude the effects of the patient-specific genetic background. Western blotting and immunofluorescence staining confirmed efficient knockout, with loss of ODAD1 expression and the absence of ciliary localization (Supplementary Fig. S6a, b). These *ODAD1* knockout cultures faithfully recapitulated the key phenotypes observed in patient-derived models, including a reduced ciliary density, decreased MCCs number, and abnormal actin remodeling (Supplementary Fig. S6c–j).

Together, these findings suggest that ODAD1 plays a dual role in supporting ciliary motility and maintaining proper actin cytoskeletal organization in the airway epithelium.

### Pharmacological actin modulation partially restores MCC formation and ciliation

**Fig. 6:**
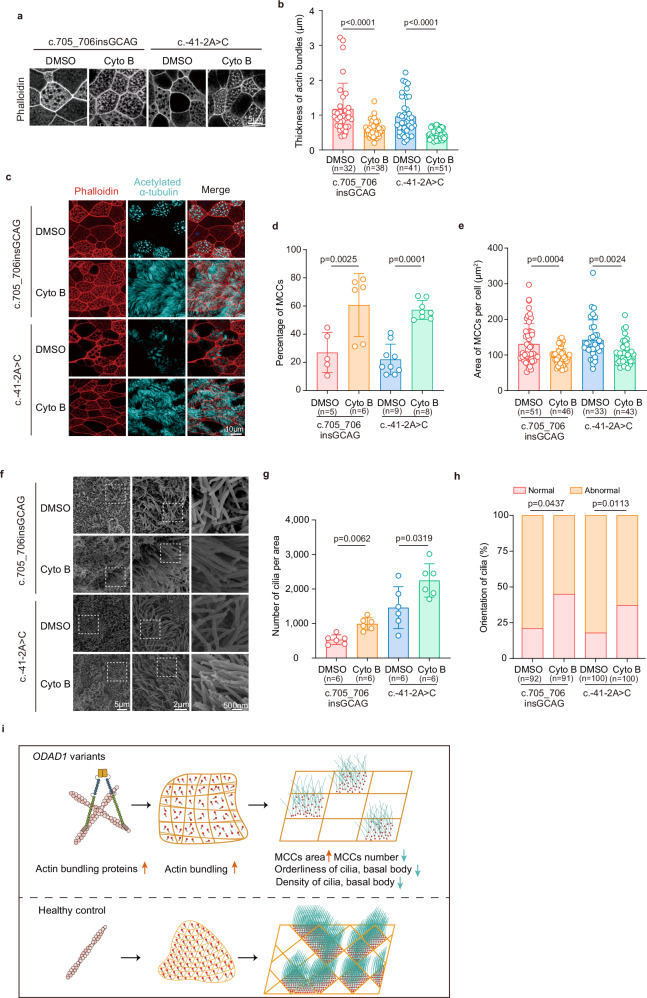
Actin inhibition restores ciliogenesis in *ODAD1*-variant ALI cultures. **a** Immunofluorescence staining for phalloidin in *ODAD1*-variant ALI cultures treated with and without cytochalasin B (Cyto B). **b** Quantification of the apical actin bundle thickness. Scatter plot showing the full width at half maximum (FWHM) of individual actin bundles measured from HIS-SIM images. Each point represents a single-bundle measurement. The data are pooled from three independent biological replicates. The number of bundles measured (n) for each condition is indicated on the *x* axis. **c**–**e** Analysis of MCC parameters following Cyto B treatment: representative images (**c**), quantification of MCC abundance (**d**), and quantification of apical surface area per MCC (**e**). **f**–**h** SEM images of cilia in *ODAD1*-variant ALI cultures treated with and without Cyto B. Representative images of the ciliary morphology are shown in **f**, while the cilia number per field and ciliary orientation are presented in **g**, **h**, respectively. **i** Working model of defective epithelial remodeling induced by *ODAD1* deficiency. (Top panel) In the airway epithelium from individuals carrying *ODAD1* variants, aberrant actin cytoskeletal remodeling (increased bundling) is associated with a reduction in the abundance of MCCs and an increase in the apical surface area of the remaining MCCs. In addition, the number of basal bodies per MCC is unchanged; the observed reduction in overall basal bodies (per field of view) is therefore a consequence of fewer MCCs being present. (Bottom panel) In contrast, the wild-type epithelium has a normal MCC abundance and apical actin organization. **b**, **d**, **e**, **g**, **h** *P* values were determined using two-tailed Student’s *t* test. Data are presented as means ± SEM. All the treatments involved the addition of 0.5 μg/mL Cyto B (or the DMSO vehicle control) to the basolateral medium on Day 12 of ALI differentiation, with all the analyses conducted at the Day 24 endpoint.

Given the pronounced actin cytoskeletal remodeling observed in *ODAD1*-variant cultures and considering that proper organization of the actin network is essential for multiciliated cell maturation by supporting basal body docking, apical surface expansion, and cilia alignment^19,20^, we tested whether the pharmacological modulation of actin dynamics could rescue MCCs-related defects. We treated *ODAD1*-variant patient-derived ALI cultures by adding the actin depolymerizing agent cytochalasin B (Cyto B; 0.5 μg/mL) to the basolateral medium for a 48-hour period beginning on Day 12 of differentiation, with the analysis performed on Day 24. Immunofluorescence staining showed that Cyto B treatment effectively reduced aberrant F-actin bundling in *ODAD1*-variant cultures (Fig. 6a, b). Notably, this effect was accompanied by a significant increase in the number of MCCs and a restoration of the apical surface area of normal MCCs (Fig. 6c–e), as well as an improved cilia density and organization (Fig. 6f–h).

These findings suggest that actin dysregulation is a key downstream consequence of *ODAD1* deficiency and contributes to impaired MCCs differentiation and ciliation while preserving the number of basal bodies per MCC (Fig. 6i). However, although Cyto B treatment increases MCCs formation and apical organization, it does not restore ciliary motility. This result is expected, as *ODAD1*-variant cells lack a functional ODA-docking complex and therefore cannot assemble outer dynein arms; this primary axonemal motor defect cannot be corrected by modulating the actin cytoskeleton (Supplementary Videos S15–S18).

### Lentivirus-mediated gene delivery rescues ciliary motility in *ODAD1*-variant organoids

**Fig. 7:**
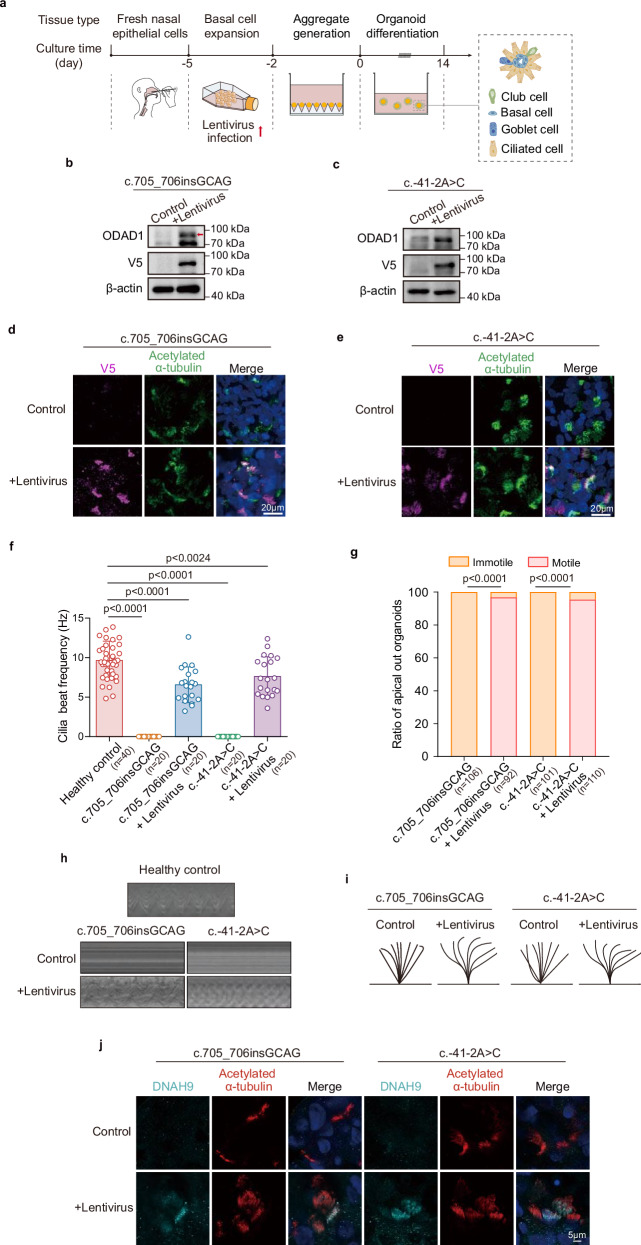
Rescue of ciliary phenotypes in *ODAD1*-variant apical-out organoids. **a** Schematic representation of the experimental design for lentiviral-mediated ODAD1 rescue in apical-out airway organoids. **b**, **c** Western blot analysis of ODAD1 expression in lentivirus-transduced *ODAD1*-variant apical-out airway organoids. **d**, **e** Immunofluorescence staining for acetylated α-tubulin (green) and V5 (magenta) in apical-out airway organoids with or without lentiviral ODAD1 expression. **f** CBF in *ODAD1*-variant apical-out airway organoids with or without lentiviral ODAD1 expression. **g** Proportion of apical-out organoids with cilia motility in the control and lentivirus-transduced groups. **h** Kymography analysis of ciliary beating in ODAD1-rescued and untreated *ODAD1*-variant apical-out organoids. **i** CBP in ODAD1-rescued and untreated *ODAD1*-variant apical-out organoids. **j** Immunofluorescence staining for acetylated α-tubulin (red) and DNAH9 (cyan) in apical-out airway organoids with or without lentiviral ODAD1 expression. All experiments involved at least three independent biological replicates and were performed on Day 14 of apical-out organoid differentiation. **f**, **g** *P* values were determined using two-tailed Student’s *t*-test, and data are presented as means ± SEM.

We introduced wild-type *ODAD1* via lentiviral transduction into apical-out organoids derived from *ODAD1*-variant patients to directly address the loss-of-function phenotypes caused by *ODAD1* variants (Fig. 7a). Western blot analysis confirmed the robust expression of the ODAD1 protein in the transduced organoids, with no expression detected in the untreated controls (Fig. 7b, c). Immunostaining further showed that the exogenous protein was correctly localized to motile cilia, indicating the successful incorporation of functional ODAD1 (Fig. 7d, e).

Functional assays using HSVM revealed that lentiviral ODAD1 re-expression restored coordinated ciliary motility, with the CBF increasing to near-physiological levels (Fig. 7f, g and Supplementary Videos S19–S22). Restored cilia exhibited a sine-wave-like beating pattern comparable to that of healthy controls (Fig. 7h, i), confirming that both the c.705_706insGCAG and c.-41-2A > C variants are loss-of-function alleles. In addition, lentiviral *ODAD1* expression rescued ODA docking in apical-out organoids, as demonstrated by the restored axonemal localization of DNAH9 (Fig. 7j), confirming ultrastructural recovery. These results indicate that targeted gene replacement effectively rescues motility defects, providing a potential avenue for therapeutic intervention in patients with *ODAD1*-related PCD.

Additionally, we also examined MCCs-related phenotypes in the apical-out organoid model to precisely confirm the effect of *ODAD1* deficiency on MCCs differentiation. The results showed a significant reduction in the abundance of MCCs (Supplementary Fig. S7a, b), an increase in the area per MCC (Supplementary Fig. S7a, c), and an abnormal actin organization (Supplementary Fig. S7d, e) in *ODAD1*-deficient organoids compared with control organoids. Importantly, lentiviral expression partially corrected the abnormal actin organization and restored the MCCs-related phenotypes (Supplementary Fig. S7f–j).

## Discussion

In this study, we identified a novel homozygous variant (c.705_706insGCAG) and a recurrent variant (c.-41-2A > C) in *ODAD1* in seven unrelated Han Chinese individuals with PCD, expanding the mutational spectrum of this ODA-docking gene. Using a combination of genomic, structural, functional, and proteomic approaches, we show that these variants result in a complete loss of ODAD1 expression, abolish ODA docking, and substantially impair ciliary motility. Surprisingly, the structural analysis revealed that different *ODAD1* variants have different effects on the axonemal architecture. In addition to these canonical structural defects, our study reveals an unexpected and novel consequence of ODAD1 dysfunction: actin cytoskeletal remodeling, which contributes to reduced MCCs abundance and compromised ciliogenesis.

*ODAD1* was first identified as a PCD gene in 2013 by Knowles et al., who reported its essential role in outer dynein arm docking. Since then, 19 *ODAD1* variants have been reported, most of which have been functionally validated as pathogenic^5,11^. The results of a cryo-EM study by Walton et al. further confirmed the complete loss of ODA and ODA-DC structures in *ODAD1*-variant respiratory organoids^12^, and a 2019 report described an expanded ciliopathy phenotype, including hearing loss, renal dysplasia, and congenital heart disease, in an individual homozygous for an *ODAD1* variant^21^. Consistent with these findings, our data indicate that the *ODAD1* variants identified here are loss-of-function alleles. Affected individuals exhibit classic PCD features, including neonatal respiratory distress, chronic wet cough, bronchiectasis, and situs inversus. TEM and cryo-EM analyses revealed a complete loss of ODAs and disrupted docking sites, and proteomics revealed the marked depletion of ODA components such as DNAH5, DNAH9, ARMC4, and TTC25. These findings mirror the defects observed in patients with other ODA-DC gene variants (e.g., ARMC4 and CCDC151)^8–10^, underscoring the essential scaffolding role of ODAD1 in ODA-DC assembly and dynein arm attachment.

In addition to its canonical role in ODA assembly, our study reveals an additional role for ODAD1 in organizing the multiciliated epithelium. *ODAD1*-deficient ALI cultures exhibited a marked reduction in the number of MCCs, together with an increase in the apical surface area of the remaining MCCs. This enlarged apical domain may arise through two nonexclusive mechanisms: (1) compensatory expansion of surviving MCCs in response to the reduced epithelial MCCs density or (2) a primary defect in apical size regulation driven by actin dysregulation, consistent with the established role of apical actin networks in constraining apical surface growth. Both possibilities are compatible with our data, and distinguishing between them will require further study. Our cytochalasin B rescue experiments suggest that actin remodeling contributes to a reduction in the number of MCCs in *ODAD1*-variant cultures. However, the mechanistic link between actin bundling and impaired MCCs abundance was not directly addressed in this study. Previous work from several groups has shown that proper actin organization is essential for the radial intercalation of nascent MCCs into the epithelial surface^19,20,22,23^, suggesting that ODAD1-associated actin defects impair this intercalation step, thereby reducing the number of fully integrated MCCs.

Another notable finding from our study is that the loss of an ODA-DC protein, despite its axonemal localization, leads to global remodeling of the actin cytoskeleton. The underlying mechanism remains unclear. Our preliminary data showing a loss of asymmetric VANGL1 localization in *ODAD1*-variant MCCs (Supplementary Fig. S8) suggest disrupted PCP polarity. Extensive evidence has indicated that PCP signaling directly coordinates the apical actin architecture, basal body docking, and MCC organization. For example, a loss of core PCP components such as Dvl or its effectors Inturned and Fuzzy significantly reduces the apical actin meshwork density and disrupts ciliogenesis in Xenopus MCCs^24,25^. These observations raise the possibility that *ODAD1* variants alter actin organization through PCP-associated pathways. However, further studies are needed to determine whether PCP disruption is a primary consequence of ODAD1 loss or a secondary effect of actin remodeling.

We treated various cultures with Cyto B, a compound known to disrupt F-actin polymerization^26^, to evaluate whether cytoskeletal dysregulation is functionally relevant to MCCs defects. Cyto B treatment alleviated abnormal actin accumulation and partially restored the number of MCCs and apical patterning. However, we acknowledge that Cyto B is a broad-spectrum agent with pleiotropic effects on actin dynamics and other cellular processes. Therefore, while its efficacy implicates actin dysregulation as a contributor to the phenotype, it cannot be used to identify the specific downstream effectors. Future work targeting individual actin-associated proteins identified in our proteomic dataset will be needed to establish a direct causal link between actin remodeling and MCCs defects. Importantly, although Cyto B increases the number of multiciliated cells, it does not rescue ciliary beating. This result is expected, as ODAD1 deficiency causes a primary defect in outer dynein arm assembly that cannot be corrected by modulating actin. Together, these findings support a dual mechanism of *ODAD1*-associated PCD: (1) a primary axonemal motor defect leads to immotile cilia and (2) a secondary actin network defect impairs MCCs differentiation and apical organization.

Importantly, lentiviral delivery of wild-type *ODAD1* restored protein expression, corrected ODA localization, and reactivated coordinated ciliary beating in differentiated organoids, confirming the causal nature of the variants. Whether lentiviral correction alone is sufficient to fully reverse both primary and secondary defects remains to be tested in vivo.

Taken together, the results of our study provide the first evidence that *ODAD1* variants disrupt motile cilia through dual mechanisms: defective dynein docking and secondary cytoskeletal remodeling. These results have important therapeutic implications. While lentiviral gene therapy appears promising for correcting the primary structural defect, pharmacological modulation of actin dynamics may represent an adjunct strategy to enhance MCCs maturation. However, the clinical utility of actin-targeting agents such as Cyto B remains uncertain because of their potential toxicity and lack of specificity. Future work should explore safer modulators or transient interventions to support mucociliary regeneration in vivo.

## Materials and methods

### Human subjects

Primary human respiratory epithelial cells were obtained from the inferior nasal turbinate of the subject via nasal scrape biopsy at Children’s Hospital, Zhejiang University School of Medicine (Hangzhou, China). Written informed consent was obtained prior to sample collection, explicitly authorizing the use of the cells for research purposes.

The protocol for this study was reviewed and approved by the Ethics Committee Review Board of Children’s Hospital Zhejiang University School of Medicine (Approval No. 2022-IRB-019-A2) and was performed in compliance with the principles of the Declaration of Helsinki.

### Collection of human airway epithelial cells

Nasal epithelial cells were collected from the inferior turbinate of participants using sterile flocked swabs with synthetic fiber tips (Kangjie Medical Devices, cat# KRD-TK01). Participants maintained a slightly reclined head position (~30° tilt) during the procedure. The swab was inserted 2–3 cm into the nasal cavity, rotated gently against the mucosal surface for 10 s, and then immediately transferred to a sterile tube containing M199 medium (Gibco, cat# 12350).

### High-speed video microscopy analysis

Ciliated respiratory epithelial cells were collected via nasal brush biopsy and suspended in prewarmed M199 medium (Gibco, cat# 12350) supplemented with 1% penicillin‒streptomycin. The cell suspensions were plated onto sterile glass-bottom culture dishes (Nest, cat# 801001) and allowed to stabilize for 30 min prior to imaging. HSVM was performed within 3 h of collection using an Olympus IX83 microscopy system equipped with a high-speed camera and differential interference contrast (DIC) optics. All the recordings were conducted at 255 frames per second with a 60×/1.42 oil immersion objective under controlled conditions of 37 °C and 5% CO₂. For the ALI samples, HSVA recordings were performed in M199 medium immediately after mucus removal, whereas nasal samples were recorded in M199 medium after minimal processing with unavoidable mucus/adherent debris, which can yield apparent “retained motility” (cilia sliding under load) despite an underlying axonemal defect.

For the quantitative analysis of cilia beating, video recordings were processed using ImageJ software (National Institutes of Health, USA). Kymographs were generated from selected regions containing active ciliary bundles using the Reslice function. The CBF was calculated by dividing the recording frame rate (255 fps) by the peak-to-peak distance measured in frames. The CBP was characterized through an analysis of the waveform amplitude and determination of the symmetry index. For each experimental condition, a minimum of 10 qualifying Supplementary Video sequences were analyzed per biological replicate. This analysis was performed across ≥ 3 independent patient samples to ensure statistical significance and reproducibility.

### Genetic analysis

Genomic DNA was extracted from peripheral blood samples using the QIAamp DNA Blood Mini Kit (Qiagen, Germany). Whole-exome sequencing was performed by BGI-Shenzhen as a certified sequencing service provider, which conducted library preparation, sequencing on Illumina NovaSeq 6000 platforms with 150 bp paired-end reads, and comprehensive bioinformatics analysis, including alignment to the GRCh38 reference genome, variant calling using their standardized pipeline, variant annotation, and initial filtering based on their established parameters. The company provided our research team with a finalized list of candidate variants that had passed their quality control and filtering criteria. Our subsequent in-house analysis included independent validation of all reported variants by Sanger sequencing, segregation analysis in available family members, a pathogenicity assessment according to ACMG-AMP guidelines, and biological interpretation of variant clinical significance in the context of PCD pathogenesis. All variant classifications and functional interpretations presented in this study were determined by our research team based on the data provided by BGI.

### Plasmid construction

The human *ODAD1* coding sequence was PCR-amplified from cDNA obtained from healthy ALI cultures using Phanta Max Master Mix (Vazyme, cat# P525-02) with primers containing BamHI/XhoI sites and an N-terminal flag or V5 tag sequence. The product was subsequently cloned and inserted into pLVX-puro (Addgene) via homologous recombination (Novoprotein, cat# NR005). The pLVX-flag-ODAD1-705_706insGCAG variant was generated by inserting GCAG into pLVX-flag-ODAD1 using site-directed mutagenesis. The specific knockout sequence targeting *ODAD1* was designed and synthesized using the *Crispr.mit.edu* website. After the primers were annealed, the sgRNA was ligated into the lenti CRISPR V2 vector (Addgene, cat# 52961) using T4 DNA ligase. The primers for the knockout of *ODAD1* are as follows: sg*ODAD1*#1 5′-CACCGAACCAGGTCAAGCGGCTTC-3′ and 5′- AAACGAAGCCGCTTGACCTGGTTC-3′; and sg*ODAD1*#2 5′-CACCGAACATGGACCGCCTGCTGAA-3′ and 5′-AAACTTCAGCAGGCGGTCCATGTTC-3′. All the constructs were verified by Sanger sequencing to ensure accurate insertion and reading frame integrity.

### Antibodies

The primary antibodies used for western blotting included rabbit anti-ODAD1 (1:1000; Atlas Antibodies, cat# HPA042524), mouse anti-FLAG (1:1000; Sigma-Aldrich, cat# F1804), mouse anti-β-actin (1:2000; Proteintech, cat# 66009-1-Ig), mouse anti-V5 (1:1000; Invitrogen, cat# R96025), and mouse anti-GAPDH (1:2000; Proteintech, cat# 60004-1-Ig) antibodies.

The following antibodies were used for immunofluorescence analyses: rabbit anti-ODAD1 (1:200; Atlas Antibodies, cat# HPA042524), mouse anti-acetylated α-tubulin (1:1000; Sigma-Aldrich, cat# T7451), rabbit anti-acetylated α-tubulin (1:1000; Cell Signaling Technology, cat# 5335), mouse anti-V5 (1:1000; Invitrogen, cat# R96025), mouse anti-CEP164 (1:50; Santa Cruz Biotechnology, cat# sc-515403), rabbit anti-VANGL1 (1:100; Cusabio, cat# CSB-PA848385ESR1HU), mouse anti-E-cadherin (1:200; Abcam, cat #ab231303), rabbit anti-SPEF2 (1:100; Atlas Antibodies, cat #HPA039606) and rabbit anti-CEP43 (Proteintech, cat# 11343-1-AP) antibodies. Alexa Fluor-conjugated antibodies (488, 568, and 647; 1:500; Invitrogen) against rabbit or mouse IgG were used as secondary antibodies.

### Human airway epithelial cell culture

Primary human airway epithelial cells (HAECs) were isolated from nasal turbinate brushings obtained from both healthy donors and PCD patients. Following collection, the cells were mechanically dissociated from the brushes and seeded in 12-well plates coated with type I collagen (Sigma-Aldrich, cat# C3867). Cells were maintained in pharmaceutical-grade culture medium consisting of DMEM/F12, 10% FBS, amphotericin, Pen/Strep, and a growth factor cocktail, as described previously for selective airway basal cell growth and expansion^27,28^. Upon reaching 80%–90% confluence, the cells were dissociated using Accutase (STEMCELL Technologies, cat#07922) for passaging or downstream applications.

### ALI culture system

The ALI system was generated using PneumaCul-ALI Medium (STEMCELL Technologies, cat# 05001) according to the manufacturer’s instructions. Briefly, HAECs were seeded onto Transwell inserts (Corning, cat# CLS3460) at a density of 2 × 10⁵ cells per insert in pharmaceutical-grade culture medium. Upon reaching 100% confluence, the apical medium was removed to establish the ALI (Day 0), while the basolateral medium was replaced with Pneumacult-ALI maintenance medium. The cultures were maintained at 37 °C with 5% CO₂, with the basolateral medium changed every 48–72 h. Functional analyses (including immunofluorescence staining, high-speed video microscopy, electron microscopy, and mass spectrometry) were performed on mature ALI cultures on Day 24 of differentiation, unless specified otherwise.

### Cytochalasin B treatment of ALI cultures

ALI cultures were treated with 0.5 μg/mL cytochalasin B (Cyto B; MedChemExpress, cat# 14930-96-2) dissolved in DMSO to perturb the actin cytoskeleton. The drug was applied to the basolateral medium for a 48-h period, starting on Day 12 of differentiation. Following treatment, the basolateral medium was replaced with fresh differentiation medium without Cyto B, and the cultures were maintained until the Day 24 endpoint for analysis. Control cultures received an equivalent volume of DMSO vehicle.

### Lentivirus production

HEK293T cells were obtained from the American Type Culture Collection (ATCC) and cultured in DMEM (Gibco, cat# 11965092) supplemented with 10% fetal bovine serum (FBS; ExCell Bio, cat# FSP500) at 37 °C in a 5% CO_2_ incubator.

Lentiviral particles were generated by co-transfecting HEK293T cells with the packaging plasmids psPAX2 (Addgene #12260), pMD2. G (Addgene #12259), and expression constructs (pLVX-ODAD1-V5) at a 2:1:4 mass ratio using PolyJet transfection reagent (SignaGen, cat# SL100688) according to the manufacturer’s protocol. Following transfection, the cells were maintained in complete DMEM for 12–16 h before the medium was replaced with fresh medium. The viral supernatant was harvested at 72 h post-transfection and clarified by centrifugation (300× *g*, 5 min, 4 °C) followed by filtration through 0.22-µm PVDF membranes (Millipore, cat# SLGV033RS). For concentration, the filtered supernatant was mixed with a PEG/NaCl solution (final concentration: 8.5% PEG8000 and 0.3 M NaCl) and incubated overnight at 4 °C with gentle rotation. The precipitate was collected by centrifugation (3000× *g*, 30 min, 4 °C), resuspended in ice-cold PBS, and aliquoted for storage at –80 °C.

### Generation of apical-out airway organoids

Apical-out airway organoids were generated using PneumaCult Apical-Out Airway Organoid Medium (STEMCELL Technologies, cat# 100-0620) according to the manufacturer’s instructions. Briefly, HAECs expanded in pharmaceutical-grade culture medium were harvested and seeded in 24-well AggreWell 400 plates (STEMCELL Technologies, cat# 34411) at a density of 200,000 cells per well in 1 mL of PneumaCult Apical-Out Airway Organoid Medium, which was prepared according to the manufacturer’s instructions. The AggreWell plate was centrifuged for 3 min at 100× *g* to sediment and aggregate the cells to the bottom of each microwell. Organoids were then incubated for 48 h at 37 °C and 5% CO_2_. After the aggregates were generated and had sufficiently matured, 1 mL of fresh PneumaCult Apical-Out Airway Organoid Medium was added to each well. The aggregates were then resuspended using a P1000 pipette and evenly distributed into three wells of a 24-well plate pre-treated with Anti-Adherence Rinsing Solution. HAECs were maintained in PneumaCult Apical-Out Airway Organoid Medium for a total of 14 days, with 50% of the medium changed every other day.

### Lentivirus transduction of apical-out organoids

For apical-out organoid transduction, 1.0 × 10^6^ patient-derived basal cells were transduced with 20 µL of the lentivirus suspension (4.35 × 10⁹ TU/mL) supplemented with polybrene (5 µg/mL) (Beyotime, cat# C0351-50 mg). Following a 72-h incubation period, transduction efficiency was assessed. The successfully transduced basal cells were then transitioned to apical-out organoid culture conditions to promote differentiation into mature organoids.

### Total RNA extraction, RT-PCR, and qRT-PCR

Total RNA was isolated from nasal cells with TRIzol (Invitrogen, cat# 1596018CN) according to the manufacturer’s instructions. DNase I was added to the isolated RNAs to avoid DNA contamination. Total RNA was then reverse transcribed into cDNA with a HiScript II 1st strand cDNA synthesis kit (Vazyme, cat# R211-01). RT-PCR was performed on a PCR Thermal Cycler (Eppendorf) with the same initial amount of cDNA, and agarose gel electrophoresis was performed on a Wide Mini-Sub Cell GT Cell (Bio-Rad). qRT-PCR analysis was performed with HiScript Q RT SuperMix (Vazyme, cat# R223-01) using a CFX-Touch System (Bio-Rad). The primers for RT-PCR of *ODAD1* in nasal cells are as follows: *ODAD1*, 5′-ACTGGGCCTTTGGAGATGGG-3′ and 5′-TTAGCCCCGGGAGTCTTTGCTGGTG-3′. The primers for qRT-PCR of *ODAD1* and *GAPDH* in nasal cells are as follows: *ODAD1*, 5′-CATACGGGCAAGACTGGAGA-3′ and 5′- AAGTTGCTTGTTGATGCGCT-3′; and *GAPDH*, 5′-GGAGCGAGATCCCTCCAAAAT-3′ and 5′-GGCTGTTGTCATACTTCTCATGG-3′.

### Western blot analysis

For Western blot assays, cells were lysed with TBSN buffer (20 mM Tris-HCl (pH 8.0), 150 mM NaCl, 0.5% NP-40, 5 mM EGTA, 1.5 mM EDTA, 0.5 mM Na_3_VO_4_, and 20 mM p-nitrophenyl phosphate) supplemented with protease inhibitor cocktail (Roche, cat# 04693132001) for 30 min on ice. After centrifugation (12,000× *g*, 15 min, 4 °C), protein concentrations were determined using a Bradford assay. Equal amounts of protein were separated by SDS-PAGE and transferred to PVDF membranes (Millipore, cat# SLGV033RS) using standard protocols. The membranes were blocked with 5% non-fat milk in TBST (Tris-buffered saline containing 0.1% Tween-20) for 1 h at room temperature and then incubated overnight at 4 °C with the appropriate primary antibodies. After three washes with TBST for 5 min each, the membranes were incubated with HRP-conjugated secondary antibodies for 1 h at room temperature, followed by three additional washes with TBST for 5 min each. The protein bands were visualized using a ChemiDoc Touch Imaging System (Bio-Rad).

### Immunofluorescence staining

Freshly isolated HAECs were suspended in culture medium and deposited onto clean glass slides, after which they were allowed to air-dry at room temperature. The cells were then fixed with 4% PFA in PBS for 15 min at room temperature. Following fixation, the samples were permeabilized and blocked with 5% bovine serum albumin (BSA) in PBSTX (PBS containing 0.1% Triton X-100) for 1 h at room temperature. Primary antibodies diluted in blocking buffer were applied and incubated overnight at 4 °C. After three 5-min washes with PBSTX, the cells were incubated with species-specific Alexa Fluor 488- or 555-conjugated secondary antibodies in blocking buffer for 1 h at room temperature in the dark. The nuclei were counterstained with DAPI (BD Biosciences, cat# 564907) following three additional PBSTX washes. Confocal imaging was performed using an Olympus FV3000 laser scanning microscope with a 60×/1.42 NA oil objective, maintaining identical acquisition parameters within each experimental set.

For immunofluorescence staining of ALI cultures, samples were fixed with 4% PFA in PBS for 4 h at room temperature. After the cells were washed with PBS, antigen retrieval was performed in 10 mM sodium citrate buffer (pH 6.0) at 95 °C for 20 min in a water bath. The samples were then permeabilized with three 30-min incubations in 0.5% PBST (PBS containing 0.5% Triton X-100) and blocked in 5% BSA prepared in 0.5% PBST for 1 h at room temperature. Primary antibodies diluted in blocking buffer were applied to the samples, which were subsequently incubated overnight at 4 °C with gentle agitation. Following three 30-min washes with 0.5% PBST, the samples were incubated for 4 h at 4 °C with species-matched Alexa Fluor-conjugated secondary antibodies, DAPI, and phalloidin (Yeasen, cat# 40734ES75). After the final washes, the ALI cultures were mounted on glass slides. Confocal imaging was performed using a Leica STELLARIS8 laser scanning microscope with a 100×/1.40 NA oil objective, maintaining identical acquisition parameters within each experimental set. Super-resolution images were randomly acquired using a CSR biotech HIS-SIM laser scanning confocal microscope at room temperature with a 100×/1.5 oil objective to capture images of the basal bodies. The acquisition settings were consistent for both the experimental and control groups within the same experiment.

For apical-out organoids, immunofluorescence assays were performed as previously described^29^. Briefly, the organoids were fixed with a 4% PFA solution for 30 min at room temperature. Following fixation, the organoids were permeabilized in 10 mL of cold (4 °C) PBT (PBS containing 0.1% Triton X-100) by gentle swirling, incubated for 10 min at 4 °C, and centrifuged at 70× *g* for 5 min at 4 °C. The resulting pellet was resuspended in cold (4 °C) OWB (PBS containing 1% BSA and 0.1% Triton X-100) for blocking and transferred to microtubes. The organoids were incubated in blocking buffer at 4 °C for 15 min. Primary antibodies, which were subsequently diluted in OWB, were added to the organoids, which were subsequently incubated overnight at 4 °C with mild agitation. After the incubation, the organoids were washed three times with OWB, each lasting 2 h at 4 °C. The organoids were subsequently incubated with species-specific Alexa fluorochrome-conjugated secondary antibodies and DAPI diluted in OWB for 3 h at room temperature in the dark. Afterward, the organoids were washed three additional times with OWB, each lasting 30 min at room temperature. For clearing, a fructose-glycerol clearing solution (minimum of 50 µL) was added using a 200-µL pipette tip with the end cut off to facilitate gentle resuspension. The organoids were incubated in the clearing solution at room temperature for 20 min. Confocal images were randomly acquired using an OLYMPUS FV3000 OSR laser scanning confocal microscope at room temperature with a 40×/1.3 oil objective. The acquisition settings were consistent for both the experimental and control groups within the same experiment.

### Transmission electron microscopy sample preparation and imaging

For the ultrastructural analysis, ALI culture-bearing polyester membranes were carefully excised and fixed overnight at 4 °C with 2.5% glutaraldehyde. Following three 15-min PBS washes, the samples were post-fixed with 1% osmium tetroxide for 1 h. Dehydration was performed through an ethanol series (30%, 50%, 70%, 80%, 90%, 95% for 15 min each; absolute ethanol for 20 min) followed by acetone treatment. Embedding was achieved through sequential infiltration: 1:1 embedding medium/acetone (1 h), 3:1 mixture (3 h), and pure embedding medium overnight at room temperature. The embedding medium was polymerized at 70 °C for 24 h. Ultrathin sections (70–90 nm) were prepared using an ultramicrotome and stained with uranyl acetate (5–10 min in 50% ethanol) and lead citrate (5–10 min).

TEM imaging was conducted on a Tecnai G2 Spirit 120 kV transmission electron microscope. Multiple fields from each sample were systematically examined to ensure representative sampling of ciliary structures.

### Scanning electron microscopy sample preparation and imaging

For the ultrastructural surface analysis, ALI culture-bearing polyester membranes were carefully excised and fixed overnight at 4 °C with 2.5% glutaraldehyde. Following three 15-min washes with PBS, the samples were post-fixed with 1% osmium tetroxide for 1 h at room temperature. Dehydration was performed through a graded ethanol series (30%, 50%, 70%, 80%, 90%, and 95% ethanol for 15 min each), followed by two 20-minute treatments with absolute ethanol. The samples were critical-point dried using a Hitachi HCP-2 system and sputter-coated with a 5-nm gold-palladium layer.

SEM imaging was performed on a Nova Nano 450 field emission scanning electron microscope. Multiple representative fields were systematically examined at magnifications ranging from 1000× to 10,000× to characterize the surface morphology of ciliated epithelial cells.

### Quantification of the cilia number

The quantification of the number of cilia per field was performed on SEM images acquired on Day 24 of ALI differentiation. All SEM images were obtained by a researcher who was blinded to the experimental groups. The cilia number was quantified in two biologically independent experiments. For each experiment, four random, non-overlapping, high-magnification (×3000) fields of view across the membrane surface were systematically analyzed. The number of cilia within the entire field of view was manually counted for each image using ImageJ software (National Institutes of Health, USA). A subset of the images was recounted by a second independent blinded researcher to ensure the reliability of the counts, and a high interobserver correlation coefficient (> 0.95) was obtained. The data are presented as the mean counts from the four technical replicate fields, which collectively represent the biological sample.

### Analysis of the ciliary orientation

The ciliary orientation was quantitatively analyzed using SEM images acquired at ×10,000 magnification from ALI cultures harvested on Day 24 of differentiation. For each ALI culture, multiple random images were captured and imported into ImageJ software. A standardized quadrant-based analysis protocol was employed to ensure an objective and unbiased assessment. The horizontal (*x*) axis of the acquired digital SEM image was uniformly set as the 0° reference axis for all the samples, providing a consistent digital coordinate framework for comparative measurements across all the experimental groups. For each image, ten non-overlapping fields of view were randomly selected using a systematic grid. Within each field, the orientation of individual cilia was determined by measuring the angle (θ) between the long axis of the axonemal shaft and the defined horizontal reference axis (0°). Each measured cilium was then categorically assigned to one of four quadrants based on its orientation: Q1 (organized, 0° ± 22.5°), Q2 (90° ± 22.5°), Q3 (180° ± 22.5°), or Q4 (270° ± 22.5°). A field of view was classified as “normal” if more than 67% of the measured cilia within it fell within the same quadrant; otherwise, it was classified as “abnormal”. The final metric for each genotype group, which is presented as the percentage of cilia with normal orientation, was calculated from the proportion of normal fields relative to the total number of fields analyzed.

### Quantification of the actin bundle thickness

High-resolution images of the apical surface of ALI cultures were acquired using high-sensitivity structured illumination microscopy (HIS-SIM). A single optical section corresponding to the apical surface of the MCCs was selected for all subsequent analyses to ensure that the measurements were performed in a consistent focal plane. Actin structures were identified based on phalloidin staining. Actin bundles were defined as continuous, linear, phalloidin-positive structures within the apical plane of MCCs to ensure objective and consistent quantification. Measurement sites were chosen at locations spatially separated by more than 1 μm from bundle terminals, branch points, or intersections with other major bundles to avoid analyzing complex regions that could distort the intensity profile.

The width of the actin bundles was quantified using the full width at half maximum (FWHM) method. For each qualified bundle, a straight line was drawn perpendicular to its long axis using ImageJ. The fluorescence intensity profile along this line was plotted, and the FWHM of the resulting intensity peak was recorded as a proxy for the bundle width. This measurement was repeated for multiple bundles per cell and multiple cells per biological replicate. All image analyses and measurements were performed by an investigator who was blinded to the experimental groups. Data pooled from at least three independent biological replicates are presented as means ± SEM, and statistical significance was determined using an unpaired two-tailed Student’s *t*-test.

### Measurement of the cortical F-actin intensity at cell-cell junctions

The fluorescence intensity of cortical F-actin at the apical boundaries of MCCs was quantified from maximum z-projections of image stacks of phalloidin-stained ALI cultures. All the images were acquired using identical laser power, gain, and exposure settings to ensure consistency. For each image, at least ten MCCs were randomly selected for analysis. Using ImageJ software, a segmented line tool was used to manually trace the apical cell perimeter, precisely following the phalloidin signal at cell-cell junctions. The mean gray value along this traced line was recorded for each cell. Background fluorescence was measured from a cell-free area of identical size adjacent to each measured cell and subtracted from the raw intensity values. The final actin junctional intensity for each genotype or condition was calculated from the averaged, background-subtracted values of all measured cells pooled from at least three independent biological replicates. All analyses were performed by an investigator who was blinded to the experimental groups.

### Label-free quantitative MS and data analysis

Differentiated airway epithelial cells were harvested from ALI cultures by gentle scraping after three PBS washes. The cell pellets were immediately flash-frozen in liquid nitrogen and stored at –80 °C until processing. For protein extraction, frozen pellets were lysed in TBSN buffer containing protease inhibitors, followed by sonication and centrifugation at 12,000× *g* for 10 min at 4 °C. Protein concentrations were determined using a BCA assay (Beyotime, cat# P0012). Aliquots containing 100 μg of protein were mixed with 20 μL of a 20 mmol/L Tris(2-carboxyethyl)phosphine (TCEP) solution, vortexed thoroughly, and incubated at 60 °C for 1 h to achieve complete reduction. Following reduction, 10 μL of a 200 mmol/L chloroacetamide (CAA) solution was added in the dark, followed by vigorous vortexing. The alkylation reaction proceeded for 30 min under light-protected conditions. For disulfide bond reduction, 5 μL of a 20 mmol/L tris(2-carboxyethyl) phosphine (TCEP) solution was added under light-protected conditions, followed by thorough vortex mixing. The reduction reaction was allowed to proceed for 15 min in the dark at room temperature (25 ± 2 °C). For protein precipitation, a 3:1 (v/v) ratio of ice-cold acetone (−20 °C pre-chilled) was added to the sample. The mixture was incubated at −20 °C for 4 h to ensure complete precipitation. Following precipitation, the samples were centrifuged at 1500× *g* for 10 min at 4 °C. The supernatant was carefully discarded, and the pellet was air-dried to remove the residual acetone. The precipitated pellet was resuspended in 50 μL of ice-cold acetone. The sample was then centrifuged at 3500× *g* for 10 min (4 °C), after which the supernatant was carefully removed, and the pellet was air-dried to remove the residual acetone. The precipitated protein pellet was resuspended in 150 μL of 0.1 mol/L ammonium bicarbonate buffer (ABB, pH 8.0). Proteolytic digestion was performed by adding 4 μL of 1 mg/mL sequencing-grade trypsin (enzyme-to-protein ratio 1:25, w/w), followed by an incubation at 37 °C for 12–16 h (overnight). Enzymatic digestion was quenched by the addition of 6 μL of 10% (v/v) trifluoroacetic acid (TFA) to achieve a final concentration of ~0.4% TFA (pH ≈3), effectively inhibiting trypsin activity. Prior to sample loading, the SOLA™ SPE cartridges were sequentially activated with 200 μL of acetonitrile (ACN) and equilibrated with 200 μL of 0.1% (v/v) trifluoroacetic acid (TFA) in ultrapure water. Each step was followed by centrifugation at 100× *g* for 1 min at 4 °C to remove the liquid phase. A 75 μL aliquot of the tryptic digest was loaded onto the preconditioned column and centrifuged at 40× *g* for 2 min at 4 °C. This loading process was repeated once to ensure maximal peptide binding. The SPE cartridge was sequentially washed with three aliquots of 100 μL of an aqueous solution containing 0.1% (v/v) trifluoroacetic acid (TFA). Each wash step was followed by centrifugation at 60× *g* for 1 min at 4 °C to remove residual contaminants. Sequential elution was performed with two 50 μL aliquots of 60% acetonitrile (ACN) containing 0.1% (v/v) trifluoroacetic acid (TFA), followed by two 50 μL aliquots of 80% ACN/0.1% TFA. Each elution step was centrifuged at 40× *g* for 2 min at 4 °C. The combined 200 μL eluate was concentrated to dryness using a vacuum centrifugal concentrator. The dried peptides were reconstituted in 10–20 μL of 0.1% (v/v) formic acid (FA) in ultrapure water, vortexed thoroughly, and centrifuged at 13,800× *g* for 10 min at 4 °C. The supernatant was carefully transferred to an LC-MS autosampler vial for analysis. Each sample was analyzed on an Orbitrap HF (Thermo) coupled to an EASY-nLC 1200 liquid chromatography (LC) system (Thermo). The LC was operated in one-column mode. The analytical column was a fused silica capillary (150 µm × 25 cm) with an integrated fritted emitter (15 µm; CoAnn Technologies) packed in-house with C18-AQ 1.9 µm resin (Thermo). The LC was equipped with two mobile phases: solvent A (0.1% formic acid, FA, in water) and solvent B (0.1% FA, 19.9% water, and 80% acetonitrile, ACN). All the solvents were of UPLC grade (Merck). Peptides were directly loaded onto the analytical column with a maximum flow rate that did not exceed the set pressure limit of 980 bar (usually ~0.6–1.0 µL/min). Peptides were subsequently separated on the analytical column using a gradient of solvent A and solvent B. The raw proteomic data and search results were deposited in the PRIDE archive to facilitate data sharing and accessibility, and are accessible through the ProteomeXchange accession number PXD064456. The exact composition of the gradient and the settings for the mass spectrometers can be found in the PRIDE archive.

The RAW spectra were transformed to mzML format by MSconvert, and the data were searched in DIA-NN (1.8.0) with label-free quantification. The MS/MS spectra data were subjected to a search of human sequences in the FASTA database. The searches allowed the oxidation of methionine residues (16 Da) and acetylation of the protein N-terminus (42 Da) as dynamic modifications. Carbamidomethylation on cysteine (57 Da) was set as the static modification. Enzyme specificity was set to “Trypsin/P specific”. The instrument type was set to Orbitrap, and the precursor mass tolerance was set to ±10 ppm (first search) and ±5 ppm (main search). The MS/MS match tolerance was set to ±0.5 Da. The peptide spectrum match FDR and the protein FDR were set to 0.01 (based on the target-decoy approach). The minimum peptide length was 5 amino acids, and the maximum amino acid length was 25. For protein quantification, unique and razor peptides were allowed. Modified peptides were allowed to be quantified. Label-free protein quantification was switched on, and unique and razor peptides were considered for quantification with a minimum ratio count of 1.

The quantification of the relative levels of the proteins was used for the differential expression analysis. Student’s *t* test was performed to determine the statistical significance of differences between groups. Proteins with adj. *P* values < 0.05 and log_2_(fold change) > 0.5 were considered to indicate significantly upregulated proteins, whereas the proteins with adj. *P* values < 0.05 and log_2_(fold change) < –0.5 were considered to indicate significantly downregulated proteins. The candidate differentially expressed proteins were subjected to a GO functional enrichment analysis using the Metascape online tool, and the network diagram of the results of the GO enrichment analysis (*P* < 0.05) was constructed using Cytoscape.

### Isolation of human respiratory cilia

Ciliary axonemes were isolated from well-differentiated ALI cultures as previously described^30^. Briefly, ciliated cell surfaces were washed twice with PBS for 5 min to remove cell debris and mucus. Ice-cold PBS was then added to both compartments of the culture dish. After 5 min of incubation on ice, the PBS solution was removed, and 200 μL of ice-cold deciliation buffer (10 mM Tris (pH 7.5), 50 mM NaCl, 10 mM CaCl_2_, 1 mM EDTA, 0.1% Triton X-100, 7 mM β-mercaptoethanol, and 1% protease inhibitor mixture (Sigma-Aldrich, cat. no. P8340)) was added to the cells in each well. After a 3-min incubation without shaking, the cilia-containing solution was transferred to a microcentrifuge tube. This procedure was repeated 10 times for efficient cilia isolation. Cellular debris and mucus were removed by centrifugation at 1000× *g* for 1 min at 4 °C. Cilia were collected by centrifuging the supernatant at 15,000× *g* for 5 min at 4 °C. The pellet was resuspended in HMKDEP buffer (30 mM HEPES (pH 7.3), 1 mM EGTA, 5 mM MgSO_4_, 0.1 mM EDTA, 25 mM NaCl, 1 mM DTT, and 1% protease inhibitor mixture) and directly used for the cryo-EM analysis.

### Negative-stain electron microscopy of human respiratory cilia

For the negative-stain electron microscopy analysis, 3 μL of purified human respiratory cilia (protein concentration 0.2–0.5 mg/mL) was applied to a glow-discharged continuous carbon-coated grid (Shanghai X-PIVOT Optoelectronic Technology, Cat# XP-CF300) and incubated for 1 min to allow sample adsorption. The excess liquid was blotted using filter paper, followed by two washes with 3 μL of a 1.5% uranyl acetate solution (Electron Microscopy Sciences, Cat# 22400). After air-drying, the samples were imaged using a Talos L120C transmission electron microscope (Thermo Fisher Scientific) operating at 120 kV with a Ceta CMOS 4 k × 4 k detector.

### Cryo-EM data collection

Isolated cilia were treated with 1% NP-40 detergent (Thermo Fisher, Cat #28324) in HMKDEP buffer at 4 °C for 30 min with gentle agitation. After centrifugation (15,000× *g*, 5 min, 4 °C), the axonemes were resuspended in HMKDEP buffer, and the A280 concentration was adjusted to 1–2 mg/mL for application to a cryo-EM grid. A 3.5 μL aliquot of the axoneme suspension was applied to glow-discharged holey carbon grids (Quantifoil, R2/1, 300 mesh gold or copper). Grids were vitrified using a Vitrobot Mark IV (Thermo Fisher Scientific) with the following parameters: 9–11 s blot time, a blot force of 5, and 100% humidity.

High-resolution images were captured using Titan Krios G4 microscopes (Thermo Fisher Scientific) operating at 300 kV and equipped with a Falcon 4 direct electron detector and Selectris X energy filter (slit width 10 eV) at the facilities of Liangzhu Laboratory (control and c.705_706insGACG) and Shuimu BioSciences (c.-41-2A > C). Images were acquired at a nominal magnification of 130,000× (pixel size 0.93 Å) under low-dose conditions (∼50 e^–^/Å^2^ total dose), with a defocus range of –1.5 to –2.5 μm. EER movie frames were collected and grouped into 40 TIFF frames using Relion_convert_to_tiff for datasets collected in Liangzhu Laboratory. Movie frames were fractioned into 32 frames of MRC format for the dataset collected in Shuimu BioSciences. The data were collected using the EPU software.

### Cryo-EM image processing of doublet microtubules

The cryo-EM data from control and variant DMTs were processed similarly using RELION 4.0^31^, as previously described^12^. Movie stacks were motion corrected using RELION’s built-in motion correction algorithm, and CTF parameters were estimated using CTFFIND4.1^32^. After preprocessing, the particles were manually picked to ensure the accurate selection of DMTs. The selected DMTs were segmented into 8-nm intervals, hereafter referred to as 8-nm particles. These particles were extracted with a box size of 768 and downscaled two times to accelerate computation. The 8-nm particles were directly subjected to an initial 8-nm 3D refinement using a previously published human respiratory doublet microtubule map (EMD-26624) as the initial model. Subsequent 3D classifications at 16-nm, 48-nm, and 96-nm intervals were performed using masks targeting specific structural features. These masks included 16-nm repeating MIPs near the inner junction, 48-nm repeating MIPs near the seam of the A tubule, and 96-nm repeating radial spokes and associated complexes. Parallel 3D classification was conducted to select good particles, and duplicated particles were removed. Density subtraction of MT and multi-reference 3D classification were performed to improve the efficiency of 3D classification when necessary. For the control and c.-41-2A > C particles, two halves of 96-nm particles were generated, and then the box size was extended to cover the whole 96 nm before a final 96-nm refinement. For the c.705_706insGCAG variant, extra densities of MIPs were observed in the 48-nm map compared to the control. Despite the application of various masks and strategies, the 48-nm map could not be further classified into 96-nm maps because of less ordered RS and IDA features.

### Statistical analysis

GraphPad Prism version 8.0 (GraphPad Software) and SPSS version 23.0 were used for statistical analyses. No data were excluded from the analysis. The sample distribution was determined using the Kolmogorov–Smirnov normality test. One-way analysis of variance (ANOVA) followed by Tukey’s post hoc test was used to evaluate the statistical significance of differences among three or more groups. Two-tailed Student’s *t* tests were used to evaluate the statistical significance of differences between two groups. Two-tailed paired-samples *t*-tests were applied to analyze the paired groups. For the nonparametric tests, the two-tailed Mann–Whitney *U* test was used to evaluate the statistical significance of differences between two groups. Two-tailed Wilcoxon matched-pairs signed-rank tests were applied to analyze the paired groups. The Kruskal–Wallis test was used to analyze differences among three or more experimental groups, followed by Dunn’s post hoc analysis. Data are shown as means ± SEM or as medians with interquartile ranges. *P* < 0.05 was considered to indicate statistical significance.

## Supplementary information

Supplementary information

Video S1. Control

Video S2. PCD-1

Video S3. PCD-2

Video S4. PCD-3

Video S5. PCD-4

Video S6. PCD-5

Video S7. PCD-7

Video S8. PCD-8

Video S9. PCD-9

Video S10. ALI-Control

Video S11. ALI-PCD-1

Video S12. ALI-PCD-3

Video S13. ALI-PCD-4

Video S14. ALI-PCD-7

Video S15. ALI-c.705_706insGCAG-DMSO

Video S16. ALI-c.705_706insGCAG-0.5CytoB

Video S17. ALI-c.-41-2A-C-DMSO

Video S18. ALI-c.-41-2A-C-0.5CytoB

Video S19. Apical-out organoids-c.705_706insGCAG

Video S20. Apical-out organoids-c.705_706insGCAG+Lentivirus

Video S21. Apical-out organoids-c.-41-2A-C

Video S22. Apical-out organoids-c.-41-2A-C+Lentivirus
