# Supplementary material for: Loss-of-function variants in ODAD1 disrupt ODA docking and induce actin cytoskeletal remodeling in primary ciliary dyskinesia
Source: Cell Discov. 2026 Mar 31;12:25. doi: 10.1038/s41421-026-00875-8 (PMC13039848; doi:10.1038/s41421-026-00875-8)
Supplement: Supplementary file 1 — Supplementary information [file 41421_2026_875_MOESM1_ESM.pdf]

Supplementary information

Supplementary Figures

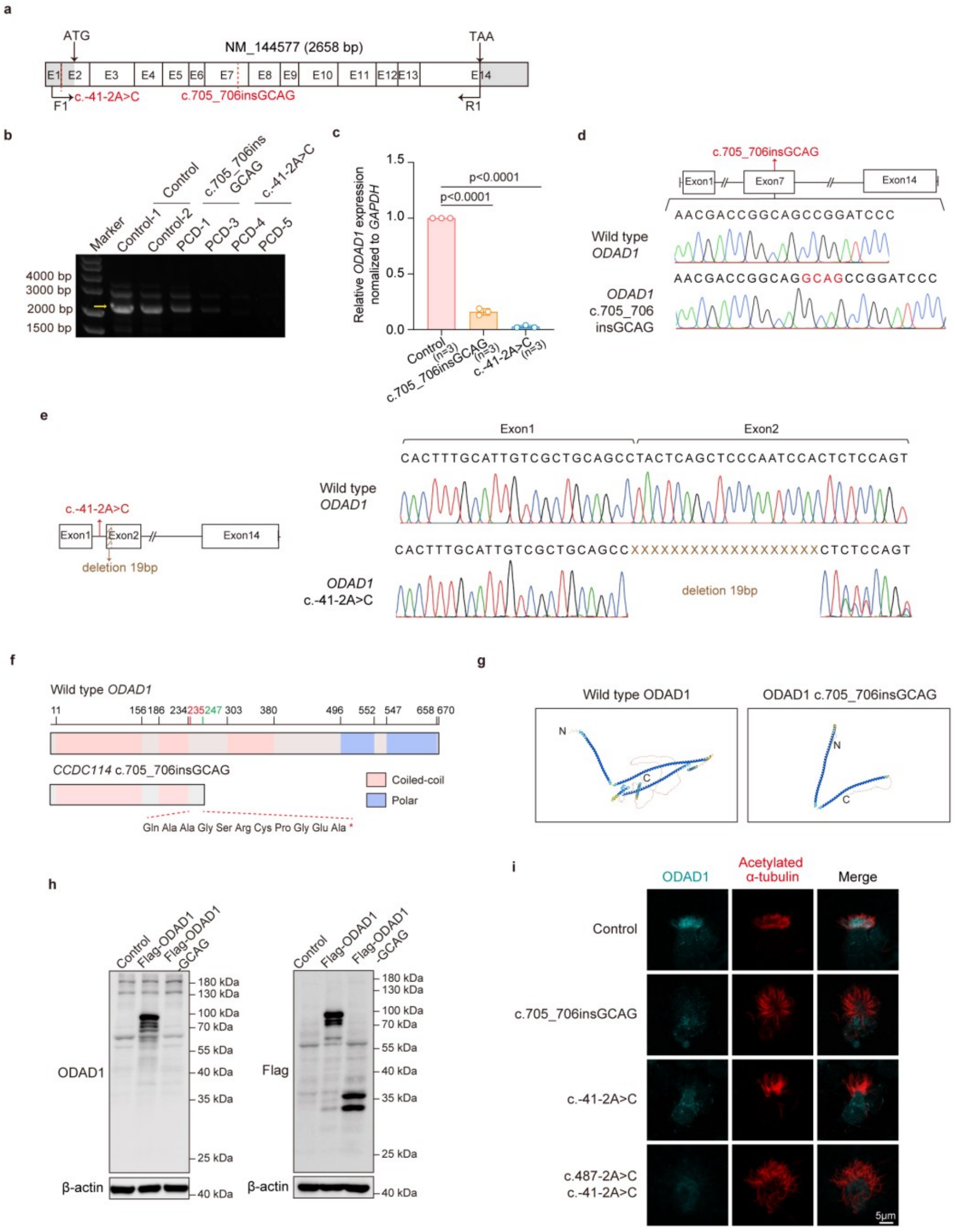

Fig. S1 Molecular consequences of *ODAD1* variants. a Schematic of the *ODAD1* transcript (NM_144577), indicating the locations of the identified c.705_706insGCAG and c.-41-2A>C variants. RT-PCR primers (F1 and R1) are located in exon 1 and exon 14, respectively. **b-c** RT-PCR analysis of *ODAD1* transcript levels in nasal epithelial cells from controls and patients with indicated variants. The expected amplicon is marked with a yellow arrow in (**b**). Quantification was normalized to *GAPDH* (**c**). **d-e** Sanger sequencing of *ODAD1* transcripts from wild-type and homozygous variant samples. **f** Schematic of the wild-type ODAD1 protein (670 amino acids) and predicted truncated variant from the

c.705_706insGCAG variant, which causes a frameshift at residue 235 and a premature stop at residue 247. **g** Predicted 3D models of the wild-type and variant ODAD1 proteins, showing retention of coiled-coil domains in the truncated form. **h** Western blot analysis of flag-tagged wild-type and variant *ODAD1* constructs expressed in hRPE-1 cells. **i** Immunofluorescence staining of acetylated α-tubulin (red) and ODAD1 (cyan) in nasal epithelial cells from controls and patients. Scale bar, 5 μm. For **c**, *P* values were determined by one-way ANOVA with Tukey's multiple comparison post-hoc test and data are presented as means ± s.e.m.

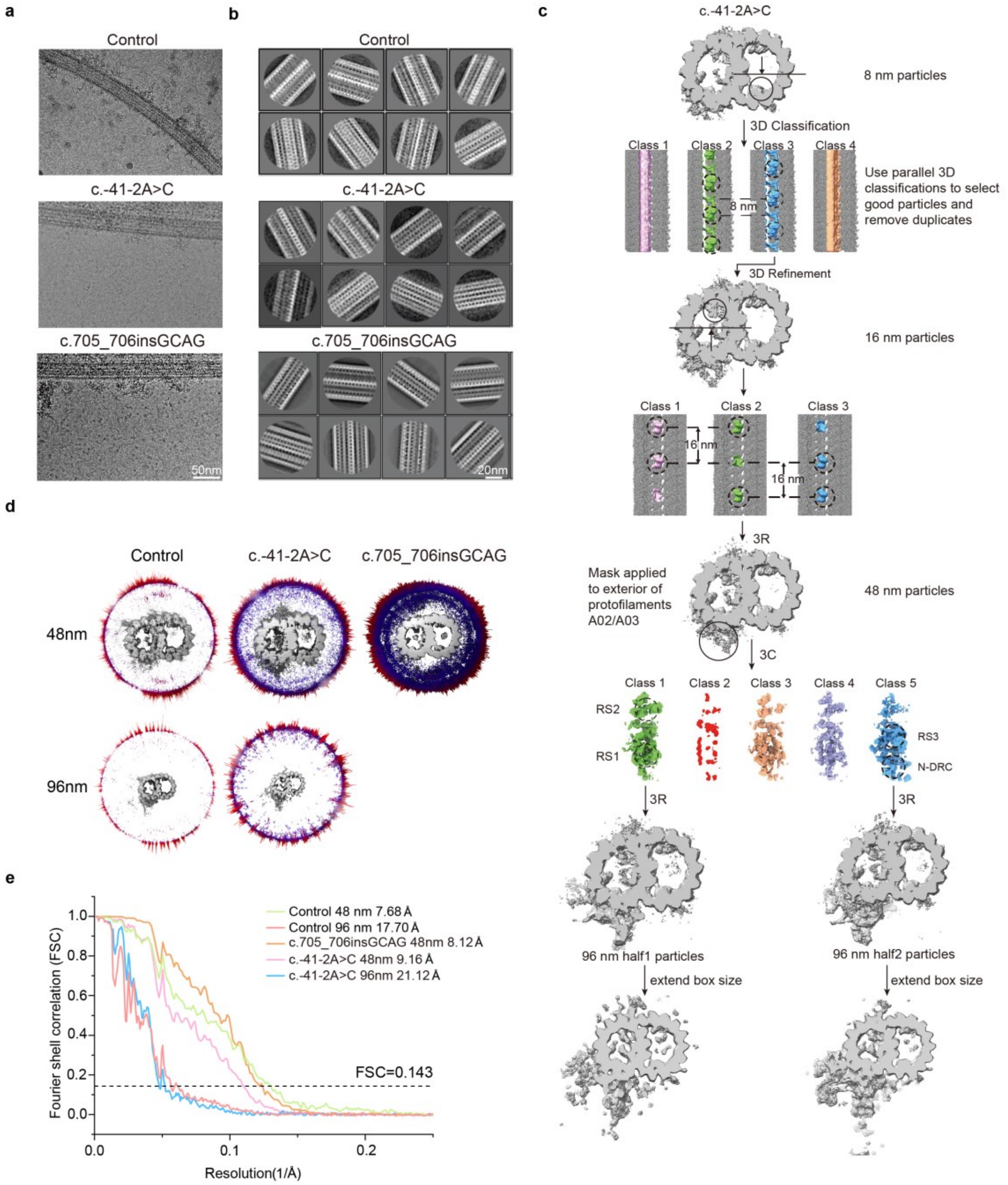

Fig. S2 Cryo-EM data processing. a Representative raw cryo-EM images of axonemes from control and *ODAD1*-variant ALI cultures. **b** Representative two-dimensional class averages of the 8-nm repeat particles extracted along the axis of the DMT. **c** The overall cryo-EM data processing scheme represented using the c.-41-2A>C variant sample. Two 96-nm maps were obtained corresponding to both halves of the 96-nm repeat: one with RS1 and RS2 and the other with RS3. 3D refinements after box size expansion yielded two equivalent maps with a 48-nm offset. The 48-nm particles of c.705_706insGCAG variant could not be further classified because of less ordered RS and IDA features. **d** Angular distribution of the particle views used for reconstruction of the 48-nm and 96-nm DMT density maps from control and *ODAD1* variants. The height of the cylinders, colored from blue to red, represents the number of particles. The final density maps of DMT are shown in gray. **e** Fourier Shell Correlation (FSC) curves for DMTs from control and *ODAD1* variants. The resolution was determined using the FSC=0.143 criterion.

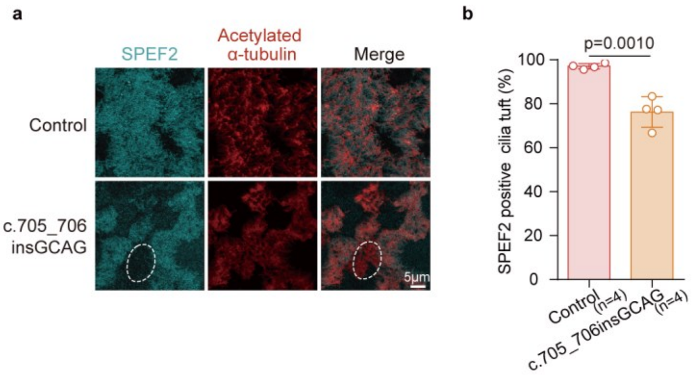

Fig. S3 Central pair (CP) related protein expression in *ODAD1*-variant ALI cultures at day 24 of differentiation. a Immunofluorescence staining of acetylated α-tubulin and central pair protein SPEF2 in ALI cultures from controls and patients. Scale bar, 5 μm. **b** Percentage of SPEF2 positive cilia tufts. Each data point represents the percentage of SPEF2 positive cilia tufts within one randomly acquired confocal image. *P* values were determined by two-tailed Student's t-test and data are presented as means ± s.e.m.

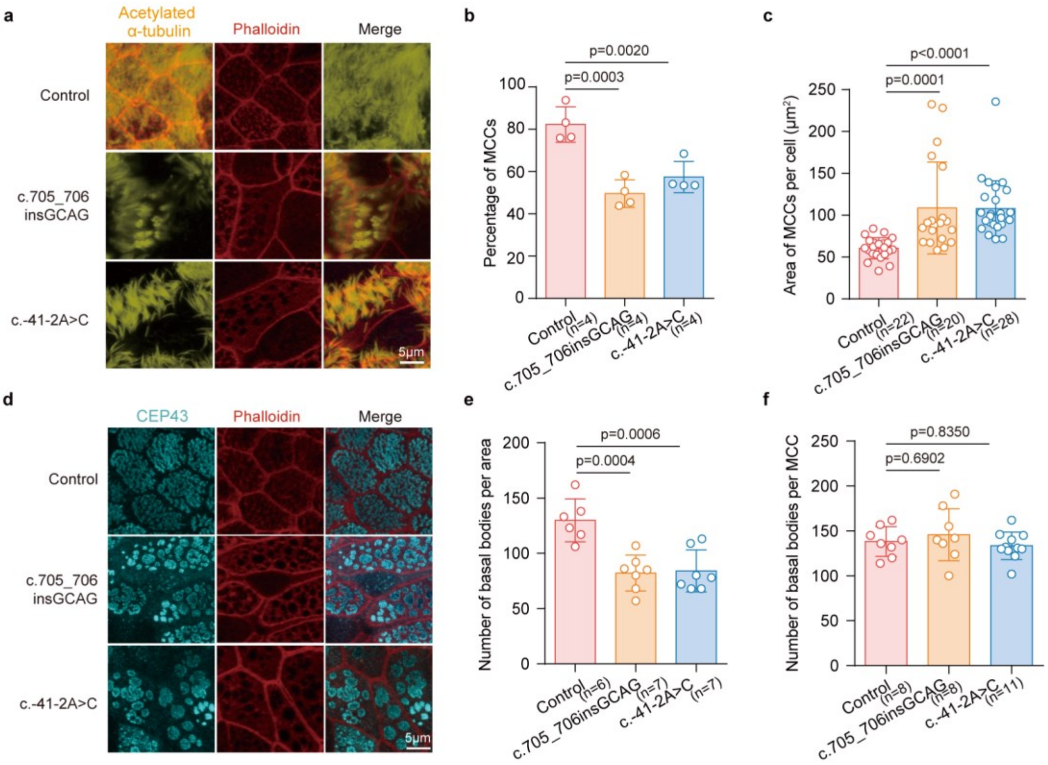

Fig. S4 MCCs-related phenotypic analysis in *ODAD1*-variant ALI cultures at day 36 of differentiation. a-c Immunofluorescence staining of acetylated α-tubulin (yellow) and phalloidin (red) in ALI cultures. (**a**) Representative images. (**b**) Percentage of MCCs at ALI apical surface. Each data point represents the percentage of MCCs within one randomly acquired confocal image. Data are from 2 control individuals (2 images per individual; total n = 4 images), 2 patients with homozygous c.705_706insGCAG (PCD-1: 2 images; PCD-3: 2 images; total n = 4 images), and 2 patients with homozygous c.-41-2A>C (PCD-4: 2 images; PCD-7: 2 images; total n = 4 images). (**c**) Area of MCCs per cell. Each data point represents the apical surface area of an individual MCC. Data are from 2 control individuals (10 and 12 MCCs per individual; total n = 22 MCCs), 2 patients with homozygous c.705_706insGCAG (PCD-1: 10 MCCs; PCD-3: 10 MCCs; total n = 20 MCCs), and 2 patients with homozygous c.-41-2A>C (PCD-4: 14 MCCs; PCD-7: 14 MCCs; total n = 28 MCCs). **d-f** Immunofluorescence staining of CEP43 (cyan) and phalloidin (red) in ALI cultures. (**d**) Representative images. (**e**) Number of basal bodies per area. Each data point represents the basal body count within a standardized area from one randomly acquired confocal field of view. Data are from 2 control individuals (3 fields per individual; total n = 6 fields), 2 patients with homozygous c.705_706insGCAG (PCD-1: 4 fields; PCD-3: 3 fields; total n = 7 fields), and 2 patients with homozygous c.-41-2A>C (PCD-4: 3 fields; PCD-7: 4 fields; total n = 7 fields). (**f**) Number of basal bodies per MCC. Each data point represents the basal body count within an individual MCC. Data are from 2 control individuals (4 MCCs per individual; total n = 8 MCCs), 2 patients with homozygous c.705_706insGCAG (PCD-1: 4 MCCs; PCD-3: 4 MCCs; total n = 8 MCCs), and 2 patients with homozygous c.-41-2A>C (PCD-4: 5 MCCs; PCD-7: 6 MCCs; total n = 11 MCCs). All experiments were performed using ALI cultures at day 36 of differentiation, with data collected from two independent culture batches. Scale bar, 5 μm. *P* values were determined by one-way ANOVA with Tukey's multiple comparison test and are indicated directly on the figures. Data are

presented as means ± s.e.m.

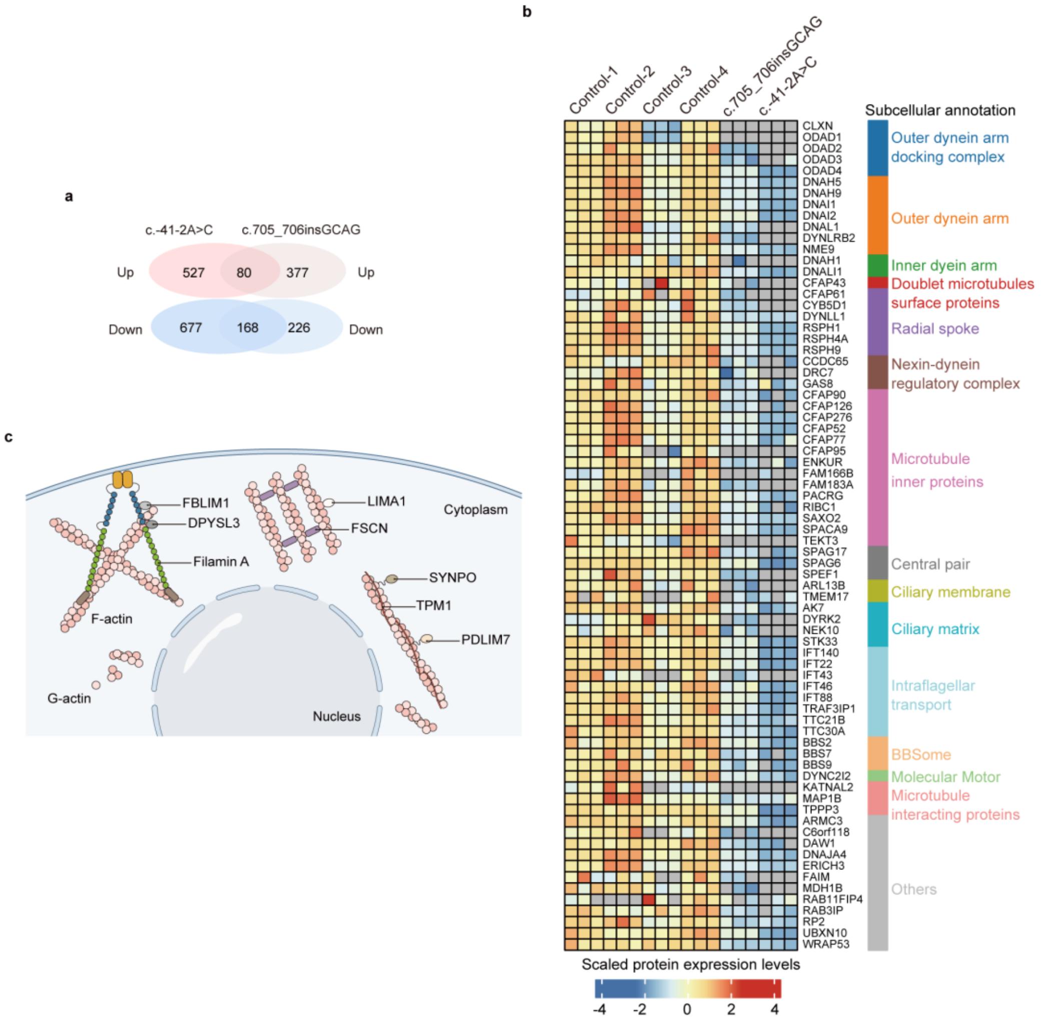

Fig. S5 Proteomics analysis of *ODAD1*-variant ALI cultures. a Venn diagram of differentially expressed proteins in *ODAD1* c.705_706insGCAG and c.-41-2A>C variant cultures. **b** Heatmap showing the expression of downregulated cilia-associated proteins in *ODAD1*-variant ALI cultures. Downregulated cilia-associated proteins were classified in term of motile cilia ultrastructural compartments. **c** Functional and interaction analysis of proteins in the "actin bundling" term of GO enrichment analysis.

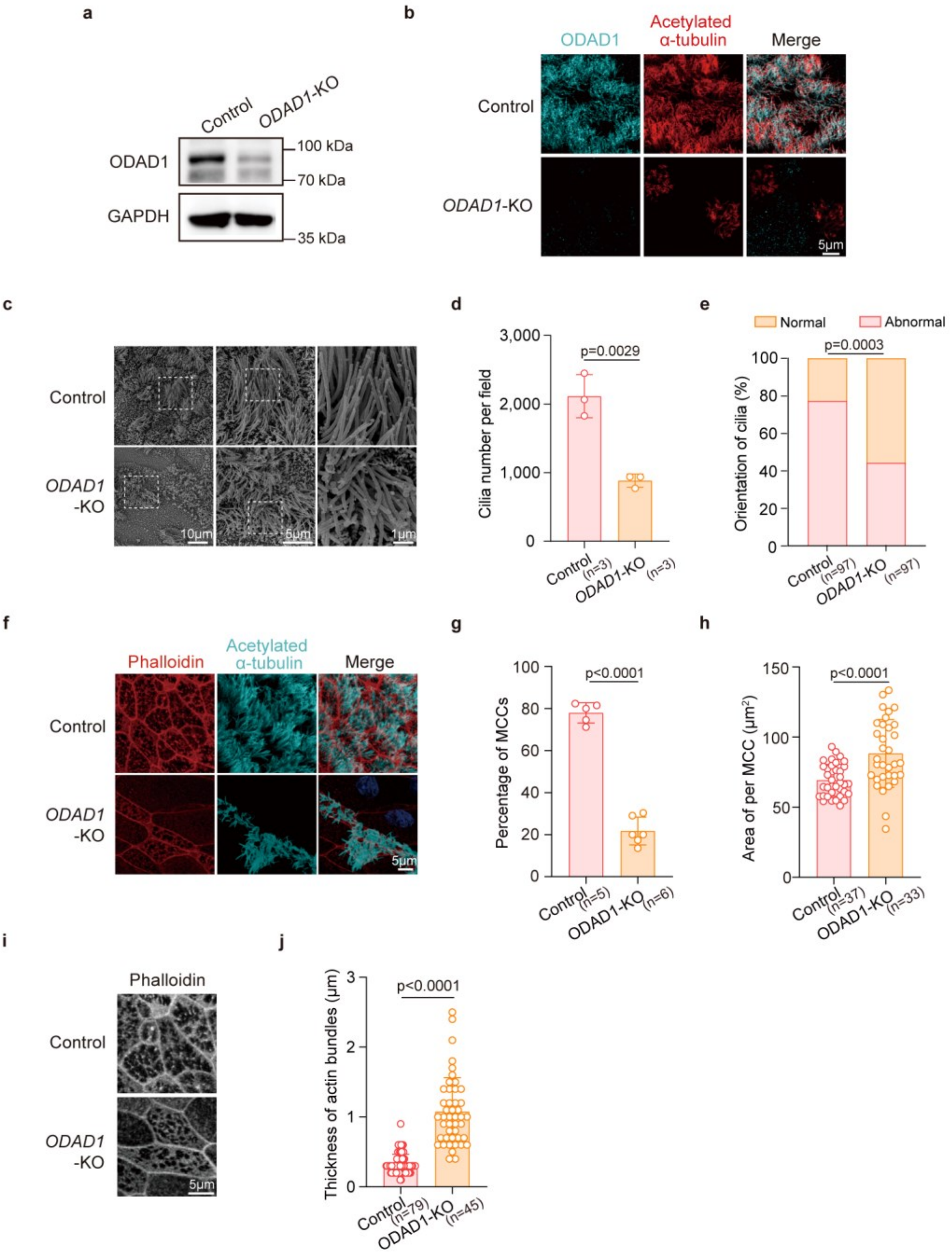

Fig. S6 MCCs-related phenotypes in *ODAD1*-knockout ALI cultures. a Western blot analysis of ODAD1 protein in control and *ODAD1*-knockout ALI cultures. **b** Immunofluorescence staining of acetylated α-tubulin (red) and ODAD1 (cyan) in control and *ODAD1*-knockout ALI cultures. **c-e** SEM of cilia. Representative ciliary morphology is shown in (**c**), with cilia number per field and ciliary orientation are presented in (**d**) and (**e**), respectively. **f-h** Immunofluorescence staining of acetylated α-tubulin (cyan) and phalloidin (red) in control and *ODAD1*-knockout ALI cultures. Representative images are shown in (**f**), the number of MCCs is presented in (**g**), and the area of MCCs per cell is shown in (**h**). **i-j** Immunofluorescence staining of F-actin in control and *ODAD1*-knockout ALI cultures. Representative images of F-actin staining in MCCs are shown in (**i**), with actin bundle thickness quantified in (j). Data are pooled from at least three independent biological replicates at Day 24 of ALI

differentiation. For **d**, **e**, **g**, **h** and **j**, *P* values were determined by two-tailed Student's t-test and data are presented as means ± s.e.m.

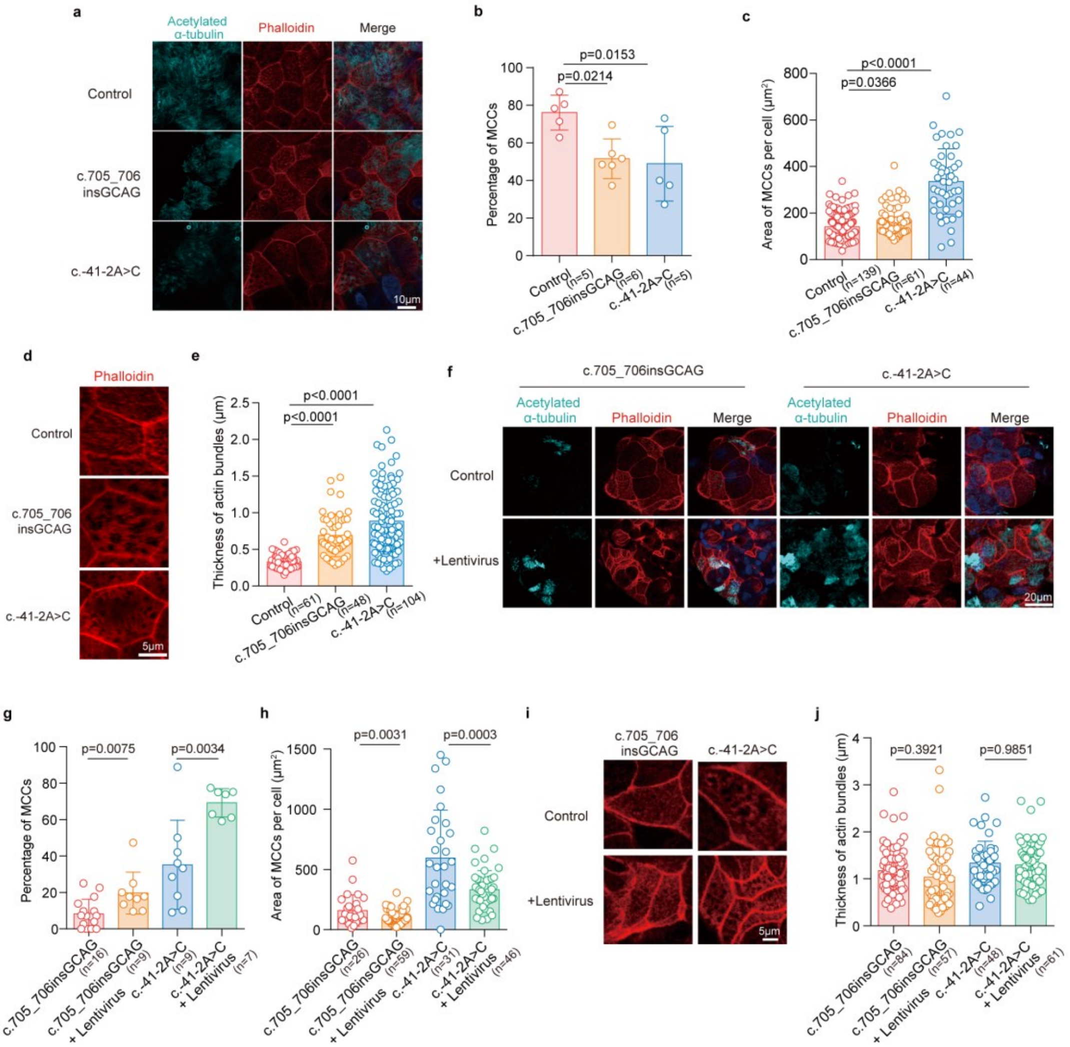

Fig. S7 MCCs-related phenotypic analysis in *ODAD1*-variant apical-out organoids. a-c Immunofluorescence staining of acetylated α-tubulin (cyan) and phalloidin (red). (**a**) Representative images. Scale bar, 10 μm. (**b**) Percentage of MCCs. Data from 2 controls (2 and 3 images per individual; total n = 5), 2 patients with homozygous c.705_706insGCAG (PCD-1: 3 images; PCD-3: 3 images; total n = 6), and 2 patients with homozygous c.-41-2A>C (PCD-4: 3 images; PCD-7: 2 images; total n = 5). (**c**) Apical surface area per MCC. Data from 2 controls (65 and 74 MCCs per individual; total n = 139), 2 patients with homozygous c.705_706insGCAG (PCD-1: 31 MCCs; PCD-3: 30 MCCs; total n = 61), and 2 patients with homozygous c.-41-2A>C (PCD-4: 22 MCCs; PCD-7: 22 MCCs; total n = 44). **d-e** Immunofluorescence staining of phalloidin. (**d**) Representative images. Scale bar, 5 μm. (**e**) Quantification of apical actin bundle thickness. Data 2 controls (30 and 31 bundles per individual; total n = 61), 2 patients with homozygous c.705_706insGCAG (PCD-1: 24 bundles; PCD-3: 24 bundles; total n = 48 bundles), and 2 patients with homozygous c.-41-2A>C (PCD-4: 44 bundles; PCD-7: 60 bundles; total n = 104 bundles). **f-h** Immunofluorescence staining of phalloidin (red) and acetylated α-tubulin (cyan) with or without lentiviral ODAD1 expression. (**f**) Representative images. Scale bar, 20 μm. (**g**) Percentage of MCCs. Data from 2 patients with homozygous c.705_706insGCAG without lentiviral ODAD1 expression (PCD-1: 8 images; PCD-3: 8 images; total n = 16), 2 c.705_706insGCAG patients

with lentiviral ODAD1 expression (PCD-1: 5 images; PCD-3: 4 images; total n = 9), 2 c.-41-2A>C patients without lentiviral ODAD1 expression (PCD-4: 4 images; PCD-7: 5 images; total n = 9), 2 c.-41-2A>C patients with lentiviral ODAD1 expression (PCD-4: 4 images; PCD-7: 3 images; total n = 7). (**h**) Area of MCCs per cell. Data are from 2 patients with homozygous c.705_706insGCAG without lentiviral ODAD1 expression (PCD-1: 13 images; PCD-3: 13 images; total n = 26), 2 c.705_706insGCAG patients with lentiviral ODAD1 expression (PCD-1: 30 images; PCD-3: 29 images; total n = 59), 2 c.-41-2A>C patients without lentiviral ODAD1 expression (PCD-4: 16 images; PCD-7: 15 images; total n = 31), 2 c.-41-2A>C patients with lentiviral ODAD1 expression (PCD-4: 23 images; PCD-7: 23 images; total n = 46). **i-j** Immunofluorescence staining of phalloidin with or without lentiviral ODAD1 expression. (**i**) Representative images. Scale bar: 5 μm. (**j**) Quantification of apical actin bundle thickness. Data are from 2 patients with homozygous c.705_706insGCAG without lentiviral ODAD1 expression (PCD-1: 40 bundles; PCD-3: 40 bundles; total n = 80), 2 c.705_706insGCAG patients with lentiviral ODAD1 expression (PCD-1: 25 bundles; PCD-3: 30 bundles; total n = 55), 2 c.-41-2A>C patients without lentiviral ODAD1 expression (PCD-4: 70 bundles; PCD-7: 70 bundles; total n = 140), 2 c.-41-2A>C patients with lentiviral ODAD1 expression (PCD-4: 120 bundles; PCD-7: 101 bundles; total n = 221). For **b** and **g**, each data point represents the percentage of MCCs within one randomly acquired confocal image. For **c** and **h**, each data point represents the apical surface area of an individual MCC. For **e** and **j**, scatter plot shows the full width at half maximum (FWHM) of individual actin bundle. The number of bundles measured (n) for each condition is indicated on the x-axis. All experiments were performed at day 14 of apical-out organoid differentiation with three independent biological replicates. *P* values were determined by one-way ANOVA with Tukey's multiple comparison test and are indicated directly on the figures. Data are presented as means ± s.e.m.

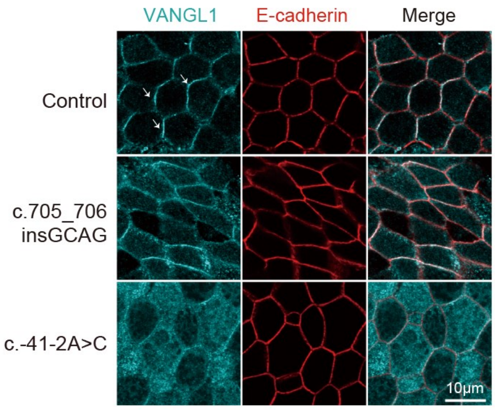

Fig. S8 Distribution of the planar cell polarity (PCP) protein VANGL1 in ALI cultures derived from control and *ODAD1*-variant individuals at day 24 of differentiation. Immunofluorescence staining for VANGL1 (cyan) and E-cadherin (red) in control and two *ODAD1*-variant ALI cultures (c.705_706insGCAG and c.-41-2A>C). Scale bar, 10 µm. White arrows indicate sites where VANGL1 exhibits distinct asymmetric enrichment at one side of the MCCs apical membrane.

Supplementary Videos Titles

Supplementary Video S1. Control

Supplementary Video S2. PCD-1

Supplementary Video S3. PCD-2

Supplementary Video S4. PCD-3

Supplementary Video S5. PCD-4

Supplementary Video S6. PCD-5

Supplementary Video S7. PCD-7

Supplementary Video S8. PCD-8

Supplementary Video S9. PCD-9

Supplementary Video S10. ALI-Control

Supplementary Video S11. ALI-PCD-1

Supplementary Video S12. ALI-PCD-3

Supplementary Video S13. ALI-PCD-4

Supplementary Video S14. ALI-PCD-7

Supplementary Video S15. ALI-c.705_706insGCAG-DMSO

Supplementary Video S16. ALI-c.705_706insGCAG-0.5CytoB

Supplementary Video S17. ALI-c.-41-2A-C-DMSO

Supplementary Video S18. ALI-c.-41-2A-C-0.5CytoB

Supplementary Video S19. Apical-out organoids-c.705_706insGCAG

Supplementary Video S20. Apical-out organoids-c.705_706insGCAG+Lentivirus

Supplementary Video S21. Apical-out organoids-c.-41-2A-C

Supplementary Video S22. Apical-out organoids-c.-41-2A-C+Lentivirus

Supplementary Table 1. Cryo-EM data collection, refinement, and validation statistics.

	Wild-type human respiratory DMT	c.705_706insGACG human respiratory DMT	c.-41-2A>C human respiratory DMT
**Data collection**			
Facility	Liangzhu	Liangzhu	Shuimu-Edison
Microscope	Titan Krios	Titan Krios	Titan Krios
Detector	Falcon 4	Falcon 4	Falcon 4
Voltage (keV)	300	300	300
Nominal magnification	130,000 x	130,000 x	130,000 x
Electron exposure ($e^-/Å^2$)	50	50	50
Defocus range set during data acquisition (μm)	-1.5 to -2.5	-1.5 to -2.5	-1.5 to -2.5
Pixel size (Å)	0.93	0.93	0.93
**Data Processing**			
Movie frames	2,593	6,752	2,806
Initial 8-nm particles	270,473	696,432	254,276
48-nm particles	13,925	168,698	27,100
96-nm particles	4,034	NA	5,310
48-nm map resolution (Å)	7.68	8.12	9.16
96-nm map resolution (Å)	17.70	NA	21.12
48-nm map accession ID	EMD-64760	EMD-64773	EMD-64775
96-nm map accession ID	EMD-64774	NA	EMD-64779

Supplementary Table 2 . Differential upregulated proteins in *ODAD1*-mutant ALI cultures

**c.705_706insGCAG**					
ProteinName	logFC	AveExpr	t	P.Value	adj.P.Val
ACOT6	1.020595967	16.28736753	5.635531181	4.26E-05	0.001096457
ADA	0.701343654	17.48606924	6.883731886	4.47E-06	0.000191274
AGR2	0.82551197	24.16044927	4.825139949	0.000205902	0.003468242
AP1S3	0.714627018	17.75853075	3.362697995	0.004130845	0.027476379
AP3S2	0.63938919	17.15003925	3.010348572	0.008565645	0.045033748
ARHGEF16	0.527246534	19.35327703	3.007645252	0.008613455	0.045217418
ASIC1	1.321123859	17.05013221	9.591280628	6.73E-08	4.26E-06
ASS1	0.542553329	22.15134258	3.393154697	0.003877603	0.026365546
C3	0.89239714	21.56834096	9.023330741	1.51E-07	8.91E-06
CBLB	4.921421711	10.98428434	54.58463212	4.44E-19	2.40E-16
CCT6B	2.085144313	18.86684256	10.99630989	1.05E-08	7.40E-07
CD109	0.563941406	18.19064234	3.105677456	0.007036594	0.039831319
CDH3	0.772777968	19.02906002	5.672992817	3.97E-05	0.001044481
CHMP7	0.572938689	16.36264526	3.38828337	0.003917043	0.026486676
COL17A1	1.088711541	19.1072207	3.330129139	0.004419877	0.028772214
CRABP2	1.198082209	20.95123981	3.639943667	0.002321816	0.018831909
CRTAP	0.913441286	16.63198218	5.169686397	0.000104352	0.002088222
DAB2IP	0.539952647	17.01408402	3.412677295	0.00372347	0.02571647
DDX21	0.531016157	18.37307187	8.19492854	5.23E-07	2.81E-05
DENND6A	0.913131184	17.24300854	5.913647296	2.53E-05	0.000755161
DGKA	0.567638735	19.68709698	3.329122543	0.004429124	0.0287829
DPYSL3	1.330549628	18.13550057	9.045857234	1.46E-07	8.69E-06
FAM83H	0.610551768	19.14697871	5.509528865	5.41E-05	0.001297263
FBLIM1	0.736640191	16.80416472	4.451979859	0.000436241	0.005973011
FLNA	0.623761463	20.85474538	3.621359283	0.002413216	0.019283764
FSCN1	0.791827989	21.10166927	4.617301497	0.000312257	0.004706642
FUT8	1.154120747	17.95925575	5.796171676	3.15E-05	0.000885036
GNPDA1	0.633525582	19.52381917	6.495508763	8.80E-06	0.000339776
GSTA4	0.862922971	18.6517909	3.794501953	0.001684612	0.015150726
IGFBP3	0.886989021	16.53577915	3.71719277	0.001977686	0.016919936
INPP5F	0.705912225	16.84977769	2.99234385	0.008889044	0.046217938
INSR	0.78308568	16.59357225	3.183606898	0.005989088	0.035829017
ITPR3	0.612917512	19.15007107	4.728035188	0.000249982	0.003999717
LAMB1	0.660794471	17.15507503	3.280970404	0.004894616	0.03090421
LDLRAP1	0.621443345	17.87415258	5.093388763	0.000121153	0.002287576
LGALS8	0.620040898	19.01428088	5.067950664	0.000127356	0.002385457
LIMA1	0.520650466	20.97434672	5.113612791	0.000116444	0.002240836
LRP1	0.623515054	17.81932177	3.886412354	0.001392533	0.013146713
LUZP1	0.567280083	17.92373633	7.218614843	2.53E-06	0.000116062

MAP3K6	0.553667548	18.12475645	3.685508648	0.002112157	0.017598806
MCAM	0.79463046	17.16607173	5.2225765	9.41E-05	0.001947581
MEMO1	0.57232552	17.41014055	4.330100427	0.000559088	0.006969589
MTO1	0.723555013	16.03312723	5.058061716	0.000129855	0.00241936
MYADM	0.60327241	17.6051233	3.469319199	0.003309996	0.023845548
NACA2	7.034918594	11.40698372	114.2384133	5.13E-24	1.80E-20
NDUFAF3	0.511324634	18.17184589	3.640386818	0.002319679	0.018831909
NLE1	0.71775256	16.42681981	3.575538606	0.002654287	0.020564476
NT5DC2	0.75513847	17.64311843	3.838583761	0.001537518	0.014135504
NXN	0.652144698	18.71007885	5.508602844	5.42E-05	0.001297263
PALM	1.055494127	18.59487822	4.223362809	0.000695486	0.00829388
PCMTD1	1.460552437	17.06775283	5.553943247	4.98E-05	0.001237818
PDLIM7	0.869697078	18.14055653	5.482504617	5.70E-05	0.001349877
PHF6	1.028875242	18.58577884	4.21178302	0.000712192	0.008446125
PLEKHG3	0.674549566	16.72753635	4.232063114	0.000683197	0.008189041
PLOD1	0.54330497	17.23920947	3.877956701	0.001417122	0.013289535
POLR1G	0.919581719	17.58296304	8.250107302	4.81E-07	2.62E-05
PPP1R13L	0.99322962	19.20698147	10.06513575	3.52E-08	2.29E-06
PRKCA	0.98998628	17.10565773	4.009617146	0.001079487	0.011149896
QPRT	1.493357599	16.38882389	4.223677537	0.000695038	0.00829388
QSOX1	0.574883373	19.47746464	3.13640619	0.006603557	0.03819155
RPL22L1	0.812133722	16.12799048	2.972079797	0.009267347	0.047583219
RPL38	0.712598468	20.68897866	4.26231494	0.000642162	0.007817238
RPS16	1.007246804	20.50709092	7.378168705	1.94E-06	9.21E-05
RPS18	1.122721054	22.67313997	10.82732972	1.30E-08	8.89E-07
RPS19	0.883441491	22.4287471	5.585093489	4.69E-05	0.001184754
SCGB1A1	1.021920879	22.54032106	3.0724726	0.007535989	0.041445091
SERPINH1	0.540643263	19.87753713	5.87445422	2.72E-05	0.000796367
SYNPO	0.83597939	16.28171184	4.991771803	0.000147964	0.002671726
TFB2M	1.236470753	17.04335642	4.483183932	0.000409478	0.005704543
TMED3	0.685720659	19.89812392	3.86600085	0.001452638	0.013568261
TMEM181	1.15376377	16.31526334	7.91775797	8.08E-07	4.18E-05
TMSB10	1.230927569	22.7971894	6.786296821	5.29E-06	0.000220968
TPM1	0.719778884	18.68791649	6.012335489	2.11E-05	0.000660541
TRIP6	0.715864611	19.55609059	3.623680029	0.002401608	0.019212862
TSC22D1	1.445260036	16.57084256	9.099276282	1.35E-07	8.26E-06
UBE3B	0.636109361	19.1675821	3.146956427	0.006461027	0.037755617
UNC13B	0.803460129	18.28002444	4.63543708	0.00030106	0.004547631
USP40	0.954498219	17.35393357	4.393406842	0.000491406	0.006368333
UTP18	0.703830315	17.39258204	5.190562274	0.000100188	0.002051655
VAV2	0.683496132	16.91818554	5.135283155	0.000111608	0.002177598
**c.-41-2A>C**					
Proteinname	logFC	AveExpr	t	P.Value	adj.P.Val

ACOT6	0.664711475	16.22366468	4.505442775	0.000391839	0.002644668
ADA	0.70897428	17.48759536	7.172952026	2.74E-06	3.78E-05
AGR2	0.624328826	24.12021265	3.648098434	0.002284311	0.010913414
AP1S3	0.740122594	17.76362986	3.498609895	0.003116339	0.014077473
AP3S2	0.707466147	17.16365464	3.252102194	0.005199187	0.021130725
ARHGEF16	0.588809579	19.36558964	3.348994893	0.00425234	0.017908847
ASIC1	1.142112729	17.01432998	8.021520048	6.88E-07	1.11E-05
ASS1	0.713498575	22.18553163	4.420447165	0.000465583	0.003010853
C3	0.688627998	21.52758713	6.964916234	3.89E-06	5.16E-05
CBLB	5.021810801	11.00436216	34.30699393	5.50E-16	7.72E-14
CCT6B	0.810319748	18.61187765	3.430990459	0.003586459	0.015712853
CD109	1.673285272	18.41251111	9.216127823	1.15E-07	2.28E-06
CDH3	0.578570895	18.9902186	4.166312131	0.000782543	0.004610573
CHMP7	0.71700402	16.39145833	4.114544147	0.000870375	0.005018591
COL17A1	1.654305259	19.22033944	5.073341421	0.000126189	0.001040168
CRABP2	1.15194508	20.94201238	3.487498716	0.003189131	0.014338841
CRTAP	1.010829391	16.86185074	3.963327057	0.001188708	0.006431662
DAB2IP	0.91030628	17.08815475	5.76398971	3.35E-05	0.000332001
DDX21	0.601809361	18.38723051	8.953455511	1.68E-07	3.17E-06
DENND6A	0.727927019	17.12741698	4.221334933	0.00069903	0.004206761
DGKA	0.75842685	19.72525461	4.445371481	0.000442595	0.002886113
DPYSL3	0.997046276	18.09492412	6.412082691	1.02E-05	0.00012205
FAM83H	0.708519629	19.16657228	6.405484457	1.04E-05	0.000123288
FBLIM1	0.823966399	17.00569957	6.388311574	1.07E-05	0.000126916
FLNA	1.254600049	20.9809131	7.271943133	2.32E-06	3.28E-05
FSCN1	0.581344883	21.05957265	3.436248238	0.003547495	0.015620097
FUT8	0.592725841	17.84697677	3.061597179	0.007710249	0.02881803
GNPDA1	0.553897814	19.50789362	5.657331298	4.10E-05	0.000394107
GSTA4	1.032600927	18.68572649	4.513199873	0.000385734	0.002612351
IGFBP3	1.41306631	16.6722251	5.488769473	5.64E-05	0.000524884
INPP5F	0.736223259	16.86077979	3.119882623	0.006836054	0.026206119
INSR	1.553805686	16.75681339	6.682343191	6.35E-06	7.98E-05
ITPR3	0.685388558	19.16456528	5.285527537	8.34E-05	0.000737926
LAMB1	1.078603858	17.24709984	5.463234739	5.93E-05	0.000545374
LDLRAP1	0.845380197	17.89847	7.185308835	2.68E-06	3.72E-05
LGALS8	0.925660844	19.07540487	7.454193098	1.71E-06	2.49E-05
LIMA1	0.509974362	20.9722115	4.981677436	0.000151143	0.001210352
LRP1	0.6575939	17.82613754	4.051996313	0.000989981	0.005596015
LUZP1	0.751218692	17.96052405	9.563965328	7.02E-08	1.46E-06
MAP3K6	0.631596278	18.1403422	4.166539692	0.000782178	0.004610573
MCAM	0.59231858	17.12560935	3.879158257	0.001414677	0.00745332
MEMO1	0.546700887	17.40501563	4.031129265	0.00103349	0.005778821
MTO1	0.60719149	16.01663928	4.164100571	0.000786105	0.004627673

MYADM	1.121792331	17.70882729	6.363002253	1.12E-05	0.00013177
NACA2	9.259426012	11.8518852	154.8777579	4.81E-26	3.38E-22
NDUFAF3	0.591263164	18.18783359	4.159557379	0.000793473	0.004667134
NLE1	0.749652935	16.47240421	3.633314746	0.00235554	0.011222503
NT5DC2	1.064225092	17.70493575	5.442681962	6.16E-05	0.00056358
NXN	0.54302722	18.68825536	3.992634528	0.001118908	0.006141516
PALM	0.705921574	18.5249637	2.821985861	0.012607525	0.042163166
PCMTD1	0.833524436	16.94234723	2.953809923	0.009625579	0.034297535
PDLIM7	0.945730108	18.15576313	5.793357403	3.17E-05	0.000317824
PHF6	0.732222205	18.52644823	2.98830848	0.008966684	0.032343615
PLEKHG3	0.815057685	16.75563798	4.937345205	0.000164985	0.001300438
PLOD1	0.748434835	17.28023544	5.367087991	7.13E-05	0.000644149
POLR1G	0.531155735	17.50527785	4.68574437	0.000272424	0.001942233
PPP1R13L	0.774454204	19.16322639	7.74230561	1.07E-06	1.65E-05
PRKCA	0.95707557	17.0874614	3.937527952	0.001253795	0.006737107
QPRT	1.307489363	16.36264898	3.691245597	0.002088554	0.010115802
QSOX1	0.806207396	19.52372944	4.387613891	0.000497739	0.003183625
RPL22L1	0.872572477	16.11007601	3.207203942	0.005706136	0.022667901
RPL38	0.513985669	20.6492561	3.095354479	0.007191361	0.027344304
RPS16	0.673161298	20.44027382	4.945184561	0.000162446	0.00128226
RPS18	0.82061636	22.61271903	7.860325956	8.88E-07	1.39E-05
RPS19	0.558490843	22.36375697	3.518032668	0.002993061	0.013676168
SCGB1A1	3.857213171	23.10737951	11.57603886	5.19E-09	1.28E-07
SERPINH1	1.206577639	20.010724	12.95643228	1.06E-09	3.05E-08
SYNPO	1.16324948	16.34716586	6.962139381	3.91E-06	5.18E-05
TFB2M	0.75273337	16.94660895	2.71514441	0.015666534	0.049943743
TMED3	0.67252127	19.89548404	3.838616182	0.001538555	0.007976007
TMEM181	0.854760158	16.27060207	5.728775835	3.58E-05	0.000351215
TMSB10	0.862349436	22.72347378	4.704268978	0.000262487	0.001894552
TPM1	1.465724256	18.83710557	12.21568996	2.45E-09	6.56E-08
TRIP6	0.553779664	19.5236736	2.791535769	0.013414753	0.044376736
TSC22D1	2.939548658	16.86970028	18.30034323	7.07E-12	3.01E-10
UBE3B	0.672454507	19.17485113	3.081297919	0.007403113	0.02798142
UNC13B	0.635771664	18.23821529	4.503646908	0.000393267	0.002644668
USP40	0.930664434	17.34916682	3.856047278	0.001484005	0.007754587
UTP18	0.502819046	17.35196112	2.841012194	0.012127305	0.040888172
VAV2	1.134363825	17.00835908	8.484537017	3.37E-07	5.86E-06

Supplementary Table 3 . Differential downregulated proteins in *ODAD1*-mutant ALI cultures

**c.705_706insGCAG**					
Proteinname	logFC	AveExpr	t	P.Value	adj.P.Val
ACSS1	-0.508366426	19.55134195	-3.531172605	0.002910678	0.022030822
AGPAT4	-0.626024529	17.29193993	-4.546815436	0.000359986	0.005170841
AK7	-0.581270489	18.1409835	-3.951968688	0.001215964	0.012046446
ALDH3B1	-0.607158462	22.22433767	-3.046338037	0.007953421	0.042939916
APH1B	-5.99542108	14.79633686	-38.66546119	8.75E-17	3.07E-14
APOBEC3A	-6.201757834	14.96140627	-14.76080091	1.63E-10	1.30E-08
ARL13B	-0.975075254	15.14863953	-4.398020816	0.000486813	0.006332173
ARMC3	-0.584291322	17.75297177	-4.428012474	0.000458006	0.006127681
ARSD	-1.2553021	15.70665886	-5.521411517	5.29E-05	0.001286486
ATP6V0A4	-0.851082192	17.85113781	-3.454225322	0.003415476	0.024241421
ATRIP	-4.974491348	13.97959308	-25.86303324	3.96E-14	6.62E-12
BBS2	-0.566421409	18.21588108	-3.229147361	0.005449907	0.033461668
BBS7	-0.725626062	16.94534404	-5.801787502	3.12E-05	0.000879307
BBS9	-0.70381534	17.10363664	-5.939340004	2.41E-05	0.000733812
BCL2L1	-0.66908725	17.83154344	-4.003597735	0.00109298	0.011180587
BSCL2	-0.966992875	18.13914578	-6.843887033	4.78E-06	0.000203637
C11orf52	-0.754898473	18.41737478	-3.996279094	0.001109617	0.011263469
C1orf87	-0.87791354	20.87173232	-4.113439388	0.000871593	0.009764071
C2CD2L	-0.82943116	16.64030585	-5.110348882	0.000117191	0.00224292
C5orf49	-0.920162115	18.05568443	-5.821924248	3.00E-05	0.000863269
C6orf118	-0.669369759	16.0254823	-3.953568327	0.001211951	0.012023647
CALB2	-5.163512901	14.13081032	-31.37194883	2.12E-15	4.97E-13
CAMKK2	-5.029711864	14.02376949	-15.89225284	5.58E-11	4.90E-09
CD38	-0.892539198	16.63584692	-4.429019541	0.000457069	0.006127681
CD55	-0.651506972	19.10940035	-4.808238003	0.000212958	0.003544582
CDKN1B	-0.570092702	16.93396945	-5.42258061	6.40E-05	0.00147158
CDS1	-0.582122249	18.15912544	-3.761410941	0.001804289	0.015853573
CEACAM7	-0.815143626	19.90522457	-6.190069108	1.52E-05	0.000519541
CES2	-0.805117118	20.4989715	-3.603947776	0.002502121	0.019810773
CFAP126	-1.016828596	18.33619773	-4.130111194	0.000842215	0.009581204
CFAP276	-0.828366754	18.30292307	-3.283074047	0.004873301	0.030867305
CFAP43	-0.640402209	15.7341056	-3.088433693	0.007291729	0.040423919
CFAP52	-0.921164327	19.54488847	-5.554061829	4.97E-05	0.001237818
CFAP61	-1.603982909	14.72924966	-5.508049428	5.43E-05	0.001297263
CFAP77	-0.877116693	18.93807439	-3.694678738	0.00207232	0.017453204
CFAP95	-4.848712102	13.87896968	-16.80609906	2.46E-11	2.24E-09
CHST6	-0.82786749	16.86322916	-5.646631672	4.17E-05	0.0010816
CKS2	-6.355969793	15.08477583	-74.05446409	4.08E-21	4.77E-18
CLCA4	-0.983389381	19.86872878	-4.424039575	0.000461719	0.006165622
CLMN	-0.506933368	19.05445958	-4.740364841	0.000243886	0.003936696
CLXN	-7.615713406	16.09257072	-22.69043167	2.84E-13	3.70E-11
CRACDL	-0.515779353	19.18615229	-4.327603613	0.000561944	0.006986014
CRIP1	-0.509852293	24.4986411	-2.968374562	0.009338203	0.047816531
CUTA	-0.963914968	17.4959569	-3.921167534	0.001295914	0.012589903
CWH43	-0.725539432	16.74893281	-5.91230667	2.54E-05	0.000755161
CYB5D1	-0.88454715	17.51152902	-4.003347313	0.001093546	0.011180587
DAW1	-1.058947879	16.63400977	-8.967526702	1.64E-07	9.50E-06
DDO	-4.90386579	13.92309263	-21.09678854	8.46E-13	9.90E-11
DEGS2	-0.950732401	17.95725841	-8.406622349	3.78E-07	2.11E-05
DGKH	-0.598552021	15.65588988	-3.278652043	0.004918214	0.030982541
DNAH1	-0.541641742	15.44857458	-3.212369171	0.005642725	0.034315584
DNAH5	-1.065574864	19.48020713	-5.108089972	0.000117711	0.002246747
DNAH9	-1.134557598	19.55400499	-3.879408979	0.001412868	0.013267355
DNAI1	-0.993622423	20.24301123	-5.628502308	4.32E-05	0.001103059
DNAI2	-0.652119453	19.11562589	-3.28304903	0.004873554	0.030867305

DNAJA4	-0.576851828	19.81319757	-4.54271226	0.000362984	0.005203271
DNAJB2	-0.855679905	18.0096856	-3.381250965	0.003974686	0.02672454
DNAL1	-0.663202203	17.4284086	-3.096715839	0.007168071	0.040032955
DNALI1	-0.917584877	21.01388161	-9.671633782	6.02E-08	3.84E-06
DRC7	-0.679224724	17.15801005	-3.685917422	0.002110365	0.017598806
DYNC2I2	-0.692270321	18.12767845	-3.354854062	0.004198689	0.027796036
DYNLL1	-0.787968542	20.79279056	-3.994500644	0.001113698	0.011288043
DYNLRB2	-1.215500894	17.77276794	-6.033162577	2.03E-05	0.000646728
DYRK2	-1.117983017	15.32317457	-5.053417127	0.000131046	0.002435091
ENKUR	-0.959194507	18.7431851	-5.383686523	6.89E-05	0.001550159
ENO4	-5.114614274	14.09169142	-26.81849792	2.29E-14	4.23E-12
ERICH3	-1.124895669	19.05401738	-3.887968602	0.001388055	0.013122072
FAHD1	-0.519923679	19.74685658	-5.180279165	0.000102217	0.002069081
FAIM	-0.734957347	16.12973255	-2.96885555	0.009328975	0.047816531
FAM166B	-7.411288118	15.92903049	-23.43996385	1.74E-13	2.36E-11
FAM183A	-1.068546371	16.71228724	-4.911165949	0.000173541	0.002994974
FSTL1	-6.457753431	15.16620274	-17.95025967	9.33E-12	9.36E-10
FUT2	-6.529036791	15.22322943	-38.0836193	1.10E-16	3.69E-14
GAS2L2	-0.731059494	18.56768745	-4.63568607	0.00030091	0.004547631
GAS8	-0.742340461	17.41803745	-4.223853942	0.000694787	0.00829388
GTF2E2	-0.560501954	18.54306894	-3.209167145	0.005680283	0.034454496
HHLA2	-5.35833928	14.28667142	-27.15788883	1.89E-14	3.59E-12
HLA-DRB1	-6.848581042	15.47886483	-17.77812913	1.08E-11	1.06E-09
HLA-DRB5	-6.767018986	15.41361519	-14.12286224	3.09E-10	2.44E-08
HOMER1	-3.86509478	13.09207582	-7.022365099	3.52E-06	0.000154626
HUS1	-4.588081998	13.6704656	-15.77065083	6.24E-11	5.41E-09
IDNK	-4.559909811	13.64792785	-32.52579577	1.22E-15	3.18E-13
IFT140	-0.576429909	19.6153726	-5.469820355	5.84E-05	0.001363148
IFT22	-0.558222796	19.60788546	-4.284673057	0.000613455	0.007519906
IFT46	-0.541970893	18.93259974	-3.251974358	0.005198012	0.032396483
IFT88	-0.527347352	19.73564766	-3.819703532	0.001598863	0.014528346
IKZF2	-0.6380594	16.74538457	-3.59059822	0.002572504	0.020121677
IL1RN	-0.837230594	21.77070585	-2.946011468	0.009777254	0.049448685
IVL	-2.222594494	20.24040353	-4.739391352	0.000244362	0.003936696
KATNAL2	-6.074539146	14.85963132	-21.60433453	5.93E-13	7.06E-11
KCT2	-0.523475814	17.77110129	-3.848160289	0.001507316	0.013967527
KLHDC9	-0.726614939	16.51666222	-6.354865405	1.13E-05	0.000413965
LIN54	-0.581789252	14.64756805	-5.161026942	0.000106131	0.002111796
LMF1	-5.899008845	14.71920708	-45.1804445	8.08E-18	3.34E-15
LRIF1	-3.714442069	12.97155365	-7.783974305	1.00E-06	5.09E-05
MAB21L4	-0.516529028	18.69896199	-3.121910702	0.006804455	0.038857313
MAP1B	-1.15471916	16.99252742	-4.152035061	0.000805108	0.009278142
MDH1B	-0.73793125	15.55985718	-3.92374082	0.001289036	0.012540424
MED16	-0.709969407	19.12962966	-4.574918213	0.000340119	0.004987469
METTL7A	-0.604612609	21.25861799	-4.435289039	0.000451283	0.006124076
MFSD11	-4.038145046	13.23051604	-13.49995335	5.89E-10	4.60E-08
MGLL	-0.78164367	19.24613183	-3.221319578	0.00553904	0.033836603
MUC13	-6.210450793	14.96836063	-25.08991578	6.26E-14	9.36E-12
NCALD	-6.361308161	15.08904653	-17.07619397	1.95E-11	1.82E-09
NEK10	-0.610319318	15.54428296	-3.895342052	0.001367035	0.012993303
NME9	-1.074972663	18.52281978	-4.485160439	0.000407841	0.005695177
NTN1	-1.548718685	15.87642396	-3.947618894	0.001226945	0.012084128
ODAD1	-8.058808636	16.44704691	-26.62466038	2.55E-14	4.49E-12
ODAD2	-1.686767087	18.74777629	-9.080097953	1.39E-07	8.36E-06
ODAD3	-1.761853422	18.82970466	-6.903498104	4.32E-06	0.000186013
ODAD4	-0.74228708	20.23440695	-6.086603815	1.84E-05	0.000601184
PACRG	-0.69511613	19.23473843	-3.582833463	0.002614351	0.020290831
PANK3	-6.597048035	15.27763843	-57.86063309	1.81E-19	1.42E-16

PIERCE2	-2.730725344	14.99181554	-6.473996646	9.15E-06	0.000349174
PTPRG	-0.830375769	14.65675087	-3.437827558	0.003533873	0.024793452
RAB11FIP4	-1.385525327	14.04473097	-3.78162836	0.00173019	0.015383357
RAB3IP	-1.0899834	14.88998921	-5.418139556	6.45E-05	0.001475718
RGS14	-0.919844669	15.14073611	-4.603215919	0.000321248	0.004800943
RIBC1	-0.638161497	17.93910601	-3.269271177	0.005014856	0.031489654
RP2	-0.518869898	19.14676937	-4.211187225	0.000713063	0.008446125
RSPH1	-0.710541033	21.20705849	-3.906927931	0.001334653	0.012841919
RSPH4A	-0.898539448	21.42117525	-4.392095622	0.000492719	0.006373594
RSPH9	-0.967125804	20.66420372	-4.588374846	0.000331007	0.004894728
RTN1	-1.747434024	16.81786752	-3.686706282	0.002106911	0.017596841
S100A7	-1.141175635	18.94333798	-3.225863183	0.005487129	0.033660781
S100A8	-1.172269062	26.16768867	-2.976704747	0.00917964	0.047271109
SAMD15	-0.761797359	17.57008508	-4.417232955	0.000468154	0.006170432
SAXO2	-1.442048263	18.44139589	-5.004780311	0.000144216	0.002617497
SLC4A2	-0.696657729	16.37805644	-4.202148347	0.000726403	0.008524236
SMPD2	-0.598155101	19.46906509	-3.21793785	0.005577992	0.033980758
SMPD3	-1.022097297	16.68192412	-4.218365756	0.000702646	0.008365059
SPACA9	-1.091022272	20.80772253	-6.523839175	8.37E-06	0.000325791
SPAG17	-0.890003	17.46998778	-6.545737495	8.05E-06	0.00031606
SPAG6	-0.505562428	20.69290288	-6.167070037	1.59E-05	0.000536522
SPEF1	-1.446301184	16.76677507	-6.78982463	5.25E-06	0.000220934
SPINK5	-2.003366161	18.28339846	-7.165682977	2.76E-06	0.00012522
SPRR1A	-2.328907089	20.48704064	-6.522320505	8.40E-06	0.000325791
SPRR3	-2.339026548	24.20946306	-6.021041361	2.08E-05	0.000654549
STEAP3	-0.909558998	18.52305334	-5.275913015	8.49E-05	0.001811925
STK33	-0.563408056	18.18736341	-5.096369368	0.000120447	0.002280374
STX11	-6.521517407	15.21721393	-11.4061423	6.35E-09	4.60E-07
SUMO2	-7.40186662	15.9214933	-26.63243211	2.54E-14	4.49E-12
SYT6	-0.564298224	15.81799997	-4.582144685	0.000335194	0.004935862
TBC1D19	-8.263021618	16.61041729	-10.98148132	1.07E-08	7.46E-07
TEKT3	-5.466071909	14.37285753	-19.61267824	2.51E-12	2.75E-10
TGM3	-1.602296667	17.93750532	-5.540261348	5.11E-05	0.001254106
TMEM17	-0.887655263	17.2175547	-3.772886707	0.001761845	0.015585893
TMPRSS11B	-3.330824793	18.07351591	-5.043762045	0.000133558	0.002475223
TNFAIP8L1	-0.662269421	16.85475172	-3.238337154	0.005347076	0.0330615
TPGS2	-4.803536029	13.84282882	-15.62101801	7.17E-11	5.99E-09
TPPP	-0.809803206	20.25382522	-5.008295686	0.00014322	0.002606152
TPPP3	-0.506015279	23.66814442	-7.376364354	1.94E-06	9.21E-05
TRAF3IP1	-0.597508222	17.99082547	-3.755116469	0.001828005	0.015975991
TSPAN1	-1.261560501	18.46379182	-4.759376733	0.000234784	0.003826274
TTC21B	-0.940874366	17.89903001	-5.312629642	7.90E-05	0.00171892
TTC30A	-0.821252858	18.17479955	-7.373232105	1.95E-06	9.21E-05
TTC33	-5.191357706	14.15308616	-20.60807751	1.20E-12	1.38E-10
TXN	-0.605122662	22.77603234	-3.754936678	0.001828687	0.015975991
UBXN10	-0.549864279	18.71992992	-3.927419545	0.001279266	0.012479955
UBXN11	-1.265202452	18.41290657	-7.349232661	2.03E-06	9.46E-05
UEVLD	-0.70106352	16.31900814	-3.863632719	0.001459779	0.013586135
WDR41	-1.006357728	15.32256903	-3.324221644	0.004474417	0.029019674
WDR54	-0.506618599	18.62869126	-3.1580091	0.006314964	0.037180477
WFDC2	-1.655676528	16.15873321	-4.300384904	0.000594066	0.007307746
WRAP53	-0.79566324	17.19726234	-9.578951708	6.85E-08	4.29E-06
YIPF1	-4.013517749	13.2108142	-12.84803381	1.19E-09	8.91E-08
YJU2	-4.138590986	13.31087279	-11.32621009	7.00E-09	5.01E-07
ZCCHC17	-0.590916338	14.64688418	-3.118159443	0.006857419	0.039096196
**c.-41-2A>C**					
Proteinname	logFC	AveExpr	t	P.Value	adj.P.Val
ACSS1	-0.909785924	19.47105805	-6.128265323	1.71E-05	0.000188157

AGPAT4	-0.913819074	17.23438102	-6.007948386	2.13E-05	0.000226676
AK7	-1.167329458	18.02377171	-6.945235616	4.03E-06	5.29E-05
ALDH3B1	-2.317480749	21.88227322	-11.59442255	5.08E-09	1.26E-07
APH1B	-0.716497286	15.85212162	-3.98825456	0.001129069	0.00619005
APOBEC3A	-6.243781063	14.99502485	-14.97754758	1.33E-10	4.58E-09
ARL13B	-5.343654576	14.27492366	-28.30109586	1.02E-14	9.34E-13
ARMC3	-1.850672672	17.4996955	-13.01231365	1.00E-09	2.88E-08
ARSD	-1.092577572	15.74134243	-3.702883262	0.002038703	0.009922253
ATP6V0A4	-8.021354244	16.4170834	-34.15961172	5.87E-16	8.09E-14
ATRIP	-4.975908698	13.98072696	-25.69063078	4.43E-14	3.08E-12
BBS2	-1.572382831	18.01468879	-8.500792362	3.29E-07	5.76E-06
BBS7	-0.967982095	16.89687283	-6.145731231	1.66E-05	0.000183336
BBS9	-1.010149659	17.04236977	-9.003730248	1.56E-07	2.97E-06
BCL2L1	-0.708405865	17.82367972	-4.502604246	0.000394098	0.002646032
BSCL2	-1.084017304	18.11574089	-6.486629705	8.96E-06	0.000108912
C11orf52	-0.839434991	18.40046748	-3.437253626	0.003540092	0.015597281
C1orf87	-2.299998706	20.58731528	-10.63155011	1.68E-08	3.88E-07
C2CD2L	-0.969762588	16.61223956	-6.101086646	1.80E-05	0.000196119
C5orf49	-1.820327667	17.87565131	-9.119317096	1.32E-07	2.56E-06
C6orf118	-6.167087817	14.93367025	-41.97242672	2.53E-17	7.72E-15
CALB2	-5.32093852	14.25675082	-35.52532194	3.23E-16	5.40E-14
CAMKK2	-5.076336766	14.06106941	-15.91180543	5.51E-11	1.99E-09
CD38	-1.307488338	16.55285709	-3.583347852	0.002613197	0.012194339
CD55	-1.342534924	18.97119476	-8.521998013	3.18E-07	5.59E-06
CDKN1B	-0.800346885	16.88791862	-6.98327757	3.77E-06	5.04E-05
CDS1	-1.27480642	18.0205886	-8.867403038	1.90E-07	3.53E-06
CEACAM7	-1.171714603	19.83391038	-8.917402924	1.77E-07	3.31E-06
CES2	-1.47724117	20.36454669	-6.550082289	8.01E-06	9.87E-05
CFAP126	-1.414647571	18.25663394	-5.049872133	0.000132143	0.001080983
CFAP276	-2.389581736	17.99068007	-9.382023646	9.06E-08	1.84E-06
CFAP43	-5.905405767	14.72432461	-32.58148221	1.21E-15	1.46E-13
CFAP52	-2.063295628	19.31646221	-11.95642257	3.31E-09	8.69E-08
CFAP61	-4.989714603	13.99177168	-17.02109669	2.06E-11	7.89E-10
CFAP77	-1.872434234	18.73901088	-5.109182726	0.000117624	0.000979922
CFAP95	-4.782243619	13.8257949	-16.91837667	2.25E-11	8.53E-10
CHST6	-0.893760377	16.85420566	-6.284261287	1.29E-05	0.000149457
CKS2	-6.304817159	15.04385373	-52.25719241	8.80E-19	4.75E-16
CLCA4	-0.821274501	19.90115175	-3.649668455	0.002276875	0.010891133
CLMN	-1.055935828	18.94465908	-9.451249322	8.22E-08	1.68E-06
CLXN	-7.615713406	16.09257072	-22.67783267	2.89E-13	1.55E-11
CRACDL	-1.112892187	19.06672972	-9.317896036	9.92E-08	2.00E-06
CRIP1	-2.519214166	24.09676873	-14.44423603	2.25E-10	7.37E-09
CUTA	-7.695729494	16.1565836	-34.90986862	4.22E-16	6.44E-14
CWH43	-1.241722199	16.64569626	-9.534244882	7.32E-08	1.52E-06
CYB5D1	-7.688438454	16.15075076	-34.80087156	4.42E-16	6.61E-14
DAW1	-1.407305758	16.56433819	-8.758219819	2.24E-07	4.07E-06
DDO	-4.823816837	13.85905347	-23.82982213	1.37E-13	7.98E-12
DEGS2	-2.512794441	17.644846	-13.33862942	7.04E-10	2.09E-08
DGKH	-0.675419805	15.64045575	-3.716510612	0.001981848	0.009697017
DNAH1	-5.556902924	14.44552234	-40.23511485	4.83E-17	1.09E-14
DNAH5	-2.272294541	19.2388632	-10.54012198	1.89E-08	4.33E-07
DNAH9	-2.737856935	19.23334512	-9.03374457	1.49E-07	2.86E-06
DNAI1	-2.641452839	19.91344515	-15.04595879	1.24E-10	4.31E-09
DNAI2	-2.820184987	18.68201279	-13.3200638	7.18E-10	2.13E-08
DNAJA4	-1.033317421	19.72190445	-7.96870036	7.48E-07	1.19E-05
DNAJB2	-0.724517474	18.03591809	-3.021139619	0.008380774	0.030687266
DNAL1	-7.561049039	16.04883923	-35.67973957	3.02E-16	5.31E-14
DNALI1	-1.772448968	20.8429088	-17.89241543	9.86E-12	4.15E-10

DRC7	-7.293854994	15.835084	-57.65424826	1.95E-19	1.71E-16
DYNC2I2	-1.420223675	17.98208778	-6.3672238	1.11E-05	0.000131356
DYNLL1	-0.9488191	20.76062045	-4.855142484	0.000194209	0.001471823
DYNLRB2	-8.015868123	16.4126945	-41.49752938	3.01E-17	8.13E-15
DYRK2	-5.546771173	14.43741694	-25.35526558	5.40E-14	3.61E-12
ENKUR	-1.000145378	18.73499493	-4.431717864	0.00045504	0.002953555
ENO4	-5.087841299	14.07027304	-27.65669265	1.45E-14	1.20E-12
ERICH3	-2.058146811	18.86736716	-7.039036396	3.43E-06	4.64E-05
FAHD1	-0.502402407	19.75036083	-4.956024241	0.000159	0.001261762
FAIM	-6.280778653	15.02462292	-26.73322236	2.43E-14	1.87E-12
FAM166B	-0.97689377	17.17113718	-2.896734482	0.010821035	0.037659131
FAM183A	-6.925996519	15.54079721	-32.4373138	1.29E-15	1.51E-13
FSTL1	-6.379612144	15.10368972	-17.81487748	1.05E-11	4.37E-10
FUT2	-0.64369795	16.32146645	-4.385503625	0.000499882	0.003191518
GAS2L2	-1.867217933	18.34045576	-11.807648	3.94E-09	1.02E-07
GAS8	-0.707966901	17.42491216	-3.17610954	0.006085586	0.023823339
GTF2E2	-0.607048422	18.53375964	-4.011112944	0.001077047	0.005955984
HHLA2	-5.35217707	14.28174166	-26.77024692	2.38E-14	1.85E-12
HLA-DRB1	-6.850993799	15.48079504	-17.65799713	1.20E-11	4.89E-10
HLA-DRB5	-7.195392448	15.75631396	-22.67147389	2.90E-13	1.55E-11
HOMER1	-3.819982858	13.05598629	-7.209503417	2.57E-06	3.61E-05
HUS1	-4.424513946	13.53961116	-17.34267438	1.56E-11	6.09E-10
IDNK	-4.559909811	13.64792785	-32.45730537	1.28E-15	1.51E-13
IFT140	-1.49418291	19.431822	-13.71440011	4.73E-10	1.45E-08
IFT22	-1.607887243	19.39795257	-12.25447811	2.34E-09	6.32E-08
IFT46	-1.593506948	18.72229253	-9.860257411	4.67E-08	1.02E-06
IFT88	-1.686086585	19.50389982	-12.12252698	2.72E-09	7.22E-08
IKZF2	-0.500450123	16.7757455	-2.798601458	0.013223088	0.043866673
IL1RN	-1.484333111	21.64128535	-5.220131534	9.47E-05	0.000824306
IVL	-2.623124251	20.16029758	-5.592699782	4.63E-05	0.000438193
KATNAL2	-6.042616984	14.83409359	-20.74801202	1.09E-12	5.23E-11
KCT2	-0.519863719	17.77182371	-4.069562449	0.000954797	0.005425195
KLHDC9	-0.565830252	16.54881916	-3.239529678	0.005336468	0.021563876
LIN54	-0.695356177	14.6484998	-3.348659163	0.004255305	0.017908847
LMF1	-5.878877079	14.70310166	-47.89792236	3.35E-18	1.19E-15
LRIF1	-3.536655832	12.82932467	-9.698182391	5.83E-08	1.25E-06
MAB21L4	-0.618572685	18.67855326	-3.713276082	0.001995197	0.009751055
MAP1B	-0.892819026	17.04490745	-3.376307153	0.004017885	0.017122334
MDH1B	-5.707443432	14.56595475	-39.50433283	6.39E-17	1.36E-14
MED16	-0.501490475	19.17132545	-3.059183183	0.007748734	0.028896023
METTL7A	-1.033984544	21.1727436	-7.443229346	1.75E-06	2.53E-05
MFSD11	-3.917709323	13.13416746	-14.15106703	3.02E-10	9.67E-09
MGLL	-1.144558817	19.1735488	-4.661118734	0.000286235	0.002016277
MUC13	-0.840919961	15.96077583	-3.071075147	0.007560954	0.028426433
NCALD	-6.244003611	14.99520289	-15.06238176	1.22E-10	4.26E-09
NEK10	-5.666346828	14.53307746	-41.00462802	3.61E-17	9.06E-15
NME9	-2.580464773	18.22172136	-10.50485056	1.98E-08	4.52E-07
NTN1	-6.215464587	14.97237167	-16.51809253	3.19E-11	1.19E-09
ODAD1	-8.058808636	16.44704691	-26.60786935	2.61E-14	1.99E-12
ODAD2	-9.085129709	17.26810377	-48.85770836	2.47E-18	1.02E-15
ODAD3	-0.770140724	19.0280472	-3.927435961	0.001280226	0.006852915
ODAD4	-2.135847808	19.95569481	-17.46855655	1.40E-11	5.60E-10
PACRG	-1.565836444	19.06059436	-7.738142274	1.08E-06	1.65E-05
PANK3	-0.515375005	16.46093808	-3.621296998	0.002415083	0.01146022
PIERCE2	-5.537960604	14.43036848	-19.11255214	3.71E-12	1.64E-10
PTPRG	-4.81246715	13.84997372	-20.07914558	1.78E-12	8.30E-11
RAB11FIP4	-1.052262397	14.18973736	-3.282251606	0.004884079	0.02009425
RAB3IP	-1.147762237	14.87843344	-4.823149461	0.000206978	0.001551338

RGS14	-1.101627164	15.09843476	-5.402287574	6.66E-05	0.000604613
RIBC1	-1.629887426	17.74076083	-7.603312347	1.34E-06	1.99E-05
RP2	-0.586686326	19.13320609	-4.854976186	0.000194273	0.001471823
RSPH1	-1.97969487	20.95322772	-10.88426413	1.22E-08	2.90E-07
RSPH4A	-2.203101336	21.16026287	-10.69771818	1.54E-08	3.60E-07
RSPH9	-1.896883803	20.47825212	-8.808922698	2.07E-07	3.82E-06
RTN1	-1.268749042	16.91360451	-2.829913651	0.012405224	0.04166518
S100A7	-1.668468999	18.83787931	-4.700526432	0.000264464	0.001899113
S100A8	-2.203280145	25.96148646	-5.591500634	4.64E-05	0.0004386
SAMD15	-2.71958597	17.17852736	-13.84949679	4.11E-10	1.27E-08
SAXO2	-2.648040108	18.20019752	-9.005022878	1.56E-07	2.97E-06
SLC4A2	-1.659937484	16.18540048	-8.534587266	3.12E-07	5.53E-06
SMPD2	-0.974072223	19.39388167	-5.344276689	7.45E-05	0.000667102
SMPD3	-0.786885431	16.72896649	-3.502261335	0.003092782	0.014013294
SPACA9	-2.634237283	20.49907953	-16.06865255	4.78E-11	1.76E-09
SPAG17	-1.390365438	17.36991529	-8.493463883	3.32E-07	5.81E-06
SPAG6	-1.722161347	20.4495831	-19.45676674	2.85E-12	1.27E-10
SPEF1	-7.056035305	15.64482824	-33.20826146	9.03E-16	1.20E-13
SPINK5	-1.032274018	18.47761689	-3.040722678	0.008049353	0.029737301
SPRR1A	-2.461172808	20.4605875	-6.833760886	4.88E-06	6.28E-05
SPRR3	-3.650404762	23.94718742	-9.393468775	8.92E-08	1.82E-06
STEAP3	-1.430349793	18.41889518	-8.371974919	4.00E-07	6.82E-06
STK33	-1.639702382	17.97210454	-14.10554395	3.16E-10	1.00E-08
STX11	-6.49239766	15.19391813	-11.19526902	8.26E-09	2.01E-07
SUMO2	-7.434181048	15.94734484	-27.67213725	1.44E-14	1.20E-12
SYT6	-5.971763399	14.77741072	-50.15683368	1.65E-18	7.25E-16
TBC1D19	-8.279595357	16.62367629	-10.98854222	1.07E-08	2.59E-07
TEKT3	-5.466071909	14.37285753	-19.59888811	2.56E-12	1.17E-10
TGM3	-2.201603358	17.81764398	-7.485237964	1.63E-06	2.38E-05
TMEM17	-0.767968528	17.23950231	-3.597401573	0.002537997	0.011904209
TMPRSS11B	-8.739680865	16.99174469	-13.24732213	7.76E-10	2.27E-08
TNFAIP8L1	-1.366092376	16.71398713	-6.364583918	1.11E-05	0.000131756
TPGS2	-4.567954314	13.65436345	-21.26730512	7.57E-13	3.69E-11
TPPP	-1.802657533	20.05525435	-10.76249455	1.42E-08	3.34E-07
TPPP3	-1.9976336	23.36982076	-27.18899613	1.88E-14	1.50E-12
TRAF3IP1	-1.469210912	17.81648493	-8.880891964	1.86E-07	3.47E-06
TSPAN1	-1.155160662	18.48507179	-4.284081881	0.000614786	0.00376429
TTC21B	-1.478922336	17.79142042	-7.983322171	7.31E-07	1.17E-05
TTC30A	-1.534723502	18.03210542	-11.56842025	5.24E-09	1.29E-07
TTC33	-4.953059344	13.96244748	-21.90488245	4.86E-13	2.49E-11
TXN	-0.544654347	22.788126	-3.373586824	0.004040648	0.017198468
UBXN10	-1.707122326	18.48847831	-11.73025995	4.32E-09	1.10E-07
UBXN11	-1.862388976	18.29346926	-10.63777226	1.67E-08	3.87E-07
UEVLD	-0.694601513	16.32030054	-3.596616621	0.002542139	0.011904209
WDR41	-5.596853476	14.47748278	-27.29285487	1.77E-14	1.43E-12
WDR54	-1.921418854	18.34573121	-10.9636393	1.10E-08	2.66E-07
WFDC2	-2.527123987	15.98895978	-7.683484824	1.18E-06	1.79E-05
WRAP53	-0.932917524	17.16981149	-10.85135491	1.27E-08	3.00E-07
YIPF1	-4.01304193	13.21043354	-13.88651486	3.96E-10	1.23E-08
YJU2	-4.090581341	13.27246507	-11.69057131	4.53E-09	1.14E-07
ZCCHC17	-0.688988346	14.65258305	-3.220536395	0.005550695	0.022275732

Supplementary Table 4 Gene Ontology (GO) enrichment of upregulated proteins in *CCDC114*-mutant ALI cultures

ONTOLOGY	ID	Description	GeneRatio	BgRatio	pvalue	p.adjust	qvalue	geneID	Count
BP	GO:0051017	actin filament bundle assembly	6/76	161/18870	4.69E-05	0.038917449	0.033776457	DPYSL3/FLNA/FSCN1/LIMA1/SYNPO/TPM1	6
BP	GO:0061572	actin filament bundle organization	6/76	165/18870	5.38E-05	0.038917449	0.033776457	DPYSL3/FLNA/FSCN1/LIMA1/SYNPO/TPM1	6
BP	GO:0032956	regulation of actin cytoskeleton organization	8/76	337/18870	6.25E-05	0.038917449	0.033776457	FLNA/FSCN1/LIMA1/LRP1/MYADM/SYNPO/TMSB10/TPM1	8
CC	GO:0032432	actin filament bundle	8/75	79/19886	5.87E-10	1.32E-07	9.64E-08	FBLIM1/FLNA/FSCN1/LIMA1/PDLIM7/SYNPO/TPM1/TRIP6	8
CC	GO:0030055	cell-substrate junction	12/75	431/19886	7.02E-08	3.14E-06	2.30E-06	COL17A1/FBLIM1/FLNA/LIMA1/LRP1/MCAM/PDLIM7/RPL38/RPS16/RPS18/RPS19/TRIP6	12
CC	GO:0005788	endoplasmic reticulum lumen	7/75	313/19886	0.000177765	0.005688468	0.004170117	C3/COL17A1/CRTAP/IGFBP3/LAMB1/QSOX1/SERPINH1	7
CC	GO:0022627	cytosolic small ribosomal subunit	3/75	41/19886	0.000495488	0.013873673	0.01017055	RPS16/RPS18/RPS19	3
CC	GO:0001726	ruffle	5/75	182/19886	0.00062491	0.015553321	0.011401871	FSCN1/LIMA1/MYADM/PDLIM7/TPM1	5
CC	GO:0005884	actin filament	4/75	118/19886	0.001034923	0.020018696	0.01467536	DPYSL3/FLNA/PDLIM7/TPM1	4
CC	GO:0031252	cell leading edge	7/75	423/19886	0.00107243	0.020018696	0.01467536	DPYSL3/FSCN1/INSR/LIMA1/MYADM/PDLIM7/TPM1	7
CC	GO:0043025	neuronal cell body	7/75	489/19886	0.002446766	0.040089436	0.029388872	ASS1/DAB2IP/FLNA/INPP5F/INSR/ITPR3/SYNPO	7

CC	GO:0032040	small-subunit processome	3/75	74/19886	0.002756398	0.040089436	0.029388872	RPS16/RPS19/UTP18	3
CC	GO:0005905	clathrin-coated pit	3/75	75/19886	0.002863531	0.040089436	0.029388872	AP1S3/INPP5F/LRP1	3
MF	GO:0003779	actin binding	8/73	438/18496	0.000324455	0.042008135	0.034217153	FLNA/FSCN1/LIMA1/PDLIM7/PLEKHG3/SYNPO/TMSB10/TPM1	8
MF	GO:0045296	cadherin binding	7/73	334/18496	0.000345022	0.042008135	0.034217153	ARHGEF16/CDH3/DAB2IP/FLNA/FSCN1/LIMA1/PPP1R13L	7
MF	GO:0051219	phosphoprotein binding	4/73	93/18496	0.000500097	0.042008135	0.034217153	CBLB/LDLRAP1/PHF6/VAV2	4
MF	GO:0015643	toxic substance binding	2/73	10/18496	0.000677393	0.042554591	0.034662261	ASS1/TMEM181	2
MF	GO:0031994	insulin-like growth factor I binding	2/73	13/18496	0.001165179	0.044879857	0.036556274	IGFBP3/INSR	2
MF	GO:0017124	SH3 domain binding	4/73	122/18496	0.001380549	0.044879857	0.036556274	CBLB/DAB2IP/DPYSL3/FUT8	4
MF	GO:0031005	filamin binding	2/73	15/18496	0.001560517	0.044879857	0.036556274	DPYSL3/FBLIM1	2
MF	GO:0019838	growth factor binding	4/73	135/18496	0.002001452	0.044879857	0.036556274	CD109/IGFBP3/INSR/RPS19	4
MF	GO:0070851	growth factor receptor binding	4/73	138/18496	0.002168219	0.044879857	0.036556274	AGR2/DAB2IP/TRIP6/VAV2	4
MF	GO:0035615	clathrin adaptor activity	2/73	18/18496	0.00225655	0.044879857	0.036556274	AP1S3/LDLRAP1	2
MF	GO:0005520	insulin-like growth factor binding	2/73	19/18496	0.002515601	0.044879857	0.036556274	IGFBP3/INSR	2

MF	GO:0140312	cargo adaptor activity	2/73	19/18496	0.002515601	0.044879857	0.036556274	AP1S3/LDLRAP1	2
MF	GO:0030276	clathrin binding	3/73	70/18496	0.00267142	0.044879857	0.036556274	AP1S3/LDLRAP1/LRP1	3

Supplementary Table 5 Gene Ontology (GO) enrichment of downregulated proteins in *ODAD1*-mutant ALI cultures

ONTOLOGY	ID	Description	GeneRatio	BgRatio	pvalue	p.adjust	qvalue	geneID	Count
BP	GO:0044782	cilium organization	45/134	407/18870	1.65E-41	2.60E-38	2.51E-38	ARL13B/BBS2/BBS7/BBS9/CCDC65/CFAP126/CFAP43/CFAP61/CLXN/DAW1/DNAH1/DNAH5/DNAI1/DNAI2/DNAL1/DRC7/DYNC2I2/DYNLL1/ERICH3/GAS8/IFT140/IFT22/IFT43/IFT46/IFT88/ODAD1/ODAD2/ODAD3/ODAD4/PIERCE2/RAB3IP/RP2/RSPH1/RSPH4A/RSPH9/SPACA9/SPAG17/SPAG6/SPEF1/TEKT3/TMEM17/TRAF3IP1/TTC21B/UBXN10/WDR54	45
BP	GO:0060271	cilium assembly	43/134	378/18870	4.06E-40	3.21E-37	3.10E-37	ARL13B/BBS2/BBS7/BBS9/CCDC65/CFAP43/CLXN/DAW1/DNAH1/DNAH5/DNAI1/DNAI2/DNAL1/DRC7/DYNC2I2/DYNLL1/ERICH3/GAS8/IFT140/IFT22/IFT43/IFT46/IFT88/ODAD1/ODAD2/ODAD3/ODAD4/PIERCE2/RAB3IP/RP2/RSPH1/RSPH4A/RSPH9/SPACA9/SPAG17/SPAG6/SPEF1/TEKT3/TMEM17/TRAF3IP1/TTC21B/UBXN10/WDR54	43
BP	GO:0001578	microtubule bundle formation	28/134	125/18870	1.27E-34	6.70E-32	6.49E-32	BBS2/CCDC65/CFAP43/CLXN/DAW1/DNAH1/DNAH5/DNAI1/DNAI2/DNAL1/DRC7/GAS2L2/GAS8/MAP1B/ODAD1/ODAD2/ODAD3/ODAD4/PIERCE2/RSPH1/RSPH4A/RSPH9/SPACA9/SPAG17/SPAG6/SPEF1/TPPP/TPPP3	28
BP	GO:0007018	microtubule-based movement	40/134	420/18870	4.19E-34	1.65E-31	1.60E-31	BBS2/CCDC65/CFAP43/CFAP52/CFAP61/CLXN/CYB5D1/DAW1/DNAH1/DNAH5/DNAH9/DNAI1/DNAI2/DRC7/DYNC2I2/DYNLL1/DYNLRB2/ENKUR/GAS2L2/GAS8/IFT140/IFT22/IFT43/IFT46/IFT88/MAP1B/NEK10/ODAD1/ODAD2/ODAD3/ODAD4/PIERCE2/RSPH4A/RSPH9/SPAG17/SPAG6/SPEF1/TEKT3/TRAF3IP1/TTC21B	40
BP	GO:0099111	microtubule-based transport	23/134	214/18870	7.94E-21	1.79E-18	1.73E-18	CFAP43/DAW1/DNAH1/DNAH5/DNAH9/DNAI1/DYNC2I2/DYNLL1/GAS8/IFT140/IFT22/IFT43/IFT46/IFT88/MAP1B/NEK10/ODAD3/ODAD4/RSPH4A/SPAG17/SPAG6/TRAF3IP1/TTC21B	23

BP	GO:00<br>06858	extracellula<br>r transport	13/1<br>34	46/18<br>870	5.37E-<br>18	9.42E-<br>16	9.12E-16	CFAP43/DAW1/DNAH1/DNAH5/DNAH9/DNAI1/GAS8/NEK10/ODAD3/ODA<br>D4/RSPH4A/SPAG17/SPAG6	13
BP	GO:00<br>01539	cilium or<br>flagellum-<br>dependent<br>cell motility	18/1<br>34	163/1<br>8870	1.07E-<br>16	1.41E-<br>14	1.36E-14	BBS2/CCDC65/CFAP43/CFAP52/CLXN/DNAH1/DNAH5/DNAH9/DNAI1/DR<br>C7/ENKUR/GAS2L2/GAS8/ODAD3/RSPH4A/RSPH9/SPAG6/TEKT3	18
BP	GO:00<br>60285	cilium-<br>dependent<br>cell motility	18/1<br>34	163/1<br>8870	1.07E-<br>16	1.41E-<br>14	1.36E-14	BBS2/CCDC65/CFAP43/CFAP52/CLXN/DNAH1/DNAH5/DNAH9/DNAI1/DR<br>C7/ENKUR/GAS2L2/GAS8/ODAD3/RSPH4A/RSPH9/SPAG6/TEKT3	18
BP	GO:00<br>07368	determinati<br>on of<br>left/right<br>symmetry	14/1<br>34	133/1<br>8870	6.21E-<br>13	6.53E-<br>11	6.32E-11	ARL13B/BBS7/CFAP52/DAW1/DNAH5/DNAI1/DNAI2/ENKUR/GAS8/IFT140<br>/ODAD2/ODAD3/ODAD4/PIERCE2	14
BP	GO:00<br>60972	left/right<br>pattern<br>formation	14/1<br>34	139/1<br>8870	1.15E-<br>12	1.13E-<br>10	1.10E-10	ARL13B/BBS7/CFAP52/DAW1/DNAH5/DNAI1/DNAI2/ENKUR/GAS8/IFT140<br>/ODAD2/ODAD3/ODAD4/PIERCE2	14
BP	GO:00<br>42073	intraciliary<br>transport	10/1<br>34	49/18<br>870	1.51E-<br>12	1.40E-<br>10	1.36E-10	DAW1/DYNC2I2/DYNLL1/IFT140/IFT22/IFT43/IFT46/IFT88/TRAF3IP1/TTC2<br>1B	10
BP	GO:00<br>60632	regulation<br>of<br>microtubule<br>-based<br>movement	9/13<br>4	55/18<br>870	1.69E-<br>10	1.21E-<br>08	1.17E-08	BBS2/CCDC65/CFAP43/CLXN/CYB5D1/GAS2L2/ODAD2/RSPH4A/TTC21B	9
BP	GO:00<br>32886	regulation<br>of<br>microtubule	14/1<br>34	258/1<br>8870	4.45E-<br>09	2.81E-<br>07	2.72E-07	BBS2/CCDC65/CDKN1B/CFAP43/CLXN/CYB5D1/GAS2L2/MAP1B/ODAD2/<br>RSPH4A/SPEF1/TPPP/TRAF3IP1/TTC21B	14

		-based process							
BP	GO:0031503	protein-containing complex localization	11/134	188/18870	1.02E-07	5.17E-06	5.01E-06	DAW1/DYNC2I2/DYNLL1/IFT140/IFT22/IFT43/IFT46/IFT88/SPAG17/TRAF3IP1/TTC21B	11
BP	GO:0007288	sperm axoneme assembly	5/134	28/18870	1.44E-06	6.77E-05	6.56E-05	BBS2/CFAP43/DNAH1/DRC7/SPAG6	5
BP	GO:0120316	sperm flagellum assembly	5/134	41/18870	1.02E-05	0.00043609	0.000422389	BBS2/CFAP43/DNAH1/DRC7/SPAG6	5
BP	GO:0061512	protein localization to cilium	6/134	72/18870	1.22E-05	0.000506287	0.00049038	ARL13B/BBS9/GAS8/IFT140/ODAD4/TTC21B	6
BP	GO:0007224	smoothened signaling pathway	7/134	148/18870	9.06E-05	0.00366474	0.003549598	ARL13B/BBS7/DYRK2/GAS8/IFT140/TMEM17/TTC21B	7
BP	GO:0007286	spermatid development	8/134	206/18870	0.000113246	0.004467548	0.004327183	BBS2/CFAP43/DNAH1/DRC7/PACRG/RSPH1/SPAG17/SPAG6	8
BP	GO:0003016	respiratory system process	4/134	39/18870	0.000164908	0.006195828	0.006001162	CFAP43/DNAH9/NEK10/ODAD4	4

BP	GO:0018149	peptide cross-linking	3/134	28/18870	0.001007284	0.03601855	0.034886889	IVL/SPRR1A/TGM3	3
BP	GO:0070507	regulation of microtubule cytoskeleton organization	6/134	161/18870	0.001027145	0.03601855	0.034886889	CDKN1B/GAS2L2/MAP1B/SPEF1/TPPP/TRAF3IP1	6
BP	GO:0060295	regulation of cilium movement involved in cell motility	3/134	29/18870	0.001117704	0.036744506	0.035590036	BBS2/CLXN/GAS2L2	3
BP	GO:1902019	regulation of cilium-dependent cell motility	3/134	29/18870	0.001117704	0.036744506	0.035590036	BBS2/CLXN/GAS2L2	3
CC	GO:0005930	axoneme	38/140	165/19886	1.19E-47	1.84E-45	1.65E-45	ARL13B/BBS7/CCDC65/CFAP126/CFAP276/CFAP43/CFAP52/CFAP61/CFAP77/CFAP95/DNAH1/DNAH5/DNAH9/DNAI1/DNAI2/DNAL1/DNALI1/DYNC2I2/ENKUR/GAS8/IFT140/ODAD1/ODAD2/ODAD3/ODAD4/PACRG/PIERCE2/RSPH1/RSPH4A/RSPH9/SAXO2/SPACA9/SPAG17/SPAG6/SPEF1/TEKT3/TRAF3IP1/WDR54	38
CC	GO:0097014	ciliary plasm	38/140	166/19886	1.54E-47	1.84E-45	1.65E-45	ARL13B/BBS7/CCDC65/CFAP126/CFAP276/CFAP43/CFAP52/CFAP61/CFAP77/CFAP95/DNAH1/DNAH5/DNAH9/DNAI1/DNAI2/DNAL1/DNALI1/DYNC2	38

								I2/ENKUR/GAS8/IFT140/ODAD1/ODAD2/ODAD3/ODAD4/PACRG/PIERCE2/RSPH1/RSPH4A/RSPH9/SAXO2/SPACA9/SPAG17/SPAG6/SPEF1/TEKT3/TRAF3IP1/WDR54	
CC	GO:0031514	motile cilium	30/140	271/19886	1.81E-27	8.64E-26	7.77E-26	AK7/ARL13B/BBS2/CCDC65/CFAP43/CFAP52/CFAP61/DAW1/DNAH1/DNAH5/DNAH9/DNAI1/DNAI2/DNALI1/DRC7/ENKUR/GAS8/IFT46/IFT88/ODAD4/PACRG/RSPH1/RSPH4A/RSPH9/SAXO2/SPACA9/SPAG17/SPAG6/SPEF1/TEKT3	30
CC	GO:0005874	microtubule	29/140	467/19886	1.87E-19	6.38E-18	5.73E-18	CFAP126/CFAP276/CFAP52/CFAP77/CFAP95/DNAH1/DNAH5/DNAH9/DNAI1/DNAI2/DNAL1/DYNLL1/DYNLRB2/ENKUR/GAS2L2/GAS8/KATNAL2/MAP1B/PACRG/PIERCE2/RGS14/SAXO2/SPACA9/SPAG17/SPAG6/SPEF1/TEKT3/TPPP/TPPP3	29
CC	GO:0036064	ciliary basal body	16/140	172/19886	8.18E-14	1.78E-12	1.60E-12	BBS2/BBS7/CCDC65/CFAP126/DAW1/DYNC2I2/GAS2L2/GAS8/IFT140/IFT46/IFT88/ODAD3/RP2/SAXO2/SPACA9/TRAF3IP1	16
CC	GO:0030990	intraciliary transport particle	7/140	24/19886	2.31E-10	3.24E-09	2.92E-09	IFT140/IFT22/IFT43/IFT46/IFT88/TRAF3IP1/TTC21B	7
CC	GO:0120293	dynein axonemal particle	4/140	20/19886	1.04E-05	0.000131385	0.000118047	DNAI1/DNAI2/DNALI1/NME9	4
CC	GO:0060170	ciliary membrane	5/140	76/19886	0.000199126	0.002163236	0.001943626	ARL13B/BBS2/BBS7/BBS9/TMEM17	5
CC	GO:0001669	acrosomal vesicle	5/140	145/19886	0.003649634	0.033548563	0.03014273	ENKUR/SPACA9/SPAG17/SPAG6/TEKT3	5
MF	GO:0045504	dynein heavy chain binding	5/135	16/18496	7.87E-08	1.42E-05	1.36E-05	DNAI1/DNAI2/DNAL1/DNALI1/DYNC2I2	5

MF	GO:0015631	tubulin binding	15/135	379/18496	1.18E-07	1.42E-05	1.36E-05	DNAL1/GAS2L2/GAS8/KATNAL2/MAP1B/NCALD/PACRG/RGS14/SAXO2/SPACA9/SPAG6/SPEF1/TPPP/TPPP3/TRAF3IP1	15
MF	GO:0045505	dynein intermediate chain binding	5/135	37/18496	6.95E-06	0.00041862	0.000400427	DNAH1/DNAH5/DNAH9/DYNLL1/DYNLRB2	5
MF	GO:0003777	microtubule motor activity	5/135	68/18496	0.000138639	0.006682418	0.006392005	DNAH1/DNAH5/DNAH9/DNAI2/DYNLRB2	5
MF	GO:0008569	minus-end-directed microtubule motor activity	3/135	17/18496	0.00023992	0.008260114	0.007901136	DNAH1/DNAH5/DNAH9	3
MF	GO:0030544	Hsp70 protein binding	4/135	51/18496	0.000519971	0.015664139	0.014983387	CDKN1B/DNAJA4/DNAJB2/PACRG	4
MF	GO:0051959	dynein light intermediate chain binding	3/135	28/18496	0.001089972	0.029187034	0.027918587	DNAH1/DNAH5/DNAH9	3
